# Novel Fluorinated Phosphorus–Sulfur Heteroatom Compounds: Synthesis and Characterization of Ferrocenyl- and Aryl-Phosphonofluorodithioic Salts, Adducts, and Esters

**DOI:** 10.3390/molecules200712175

**Published:** 2015-07-03

**Authors:** Guoxiong Hua, Junyi Du, Brian A. Surgenor, Alexandra M. Z. Slawin, J. Derek Woollins

**Affiliations:** EaStCHEM School of Chemistry, University of St. Andrews, St Andrews, Fife KY16 9ST, UK; E-Mails: gh15@st-and.ac.uk (G.H.); jd83@st-and.ac.uk (J.D.); basurgenor@gmail.com (B.A.S.); amzs@st-and.ac.uk (A.M.Z.S.)

**Keywords:** 2,4-diferrocenyl-1,3,2,4-diathiadiphosphetane 2,4-disulfide, salts of phenyldithio-fluorophosphinic acids, mono- and di-halogenated alkanes, esters of phenylphosphono-fluoridodithioates

## Abstract

A series of novel ferrocenyl- and aryl-phosphonofluorodithioic salts, adducts, and esters has been prepared. The reaction of 2,4-diferrocenyl-1,3,2,4-diathiadiphosphetane 2,4-disulfide {[FcP(μ-S)S]_2_, **FcLR**} with dry KF or tetrabutylammonium fluoride (TBAF) led to the corresponding potassium and tetrabutylammonium salts of ferrocenyldithiofluorophosphinic acids. Treating potassium ferrocenyldithiofluorophosphinic acid with an equimolar amount of tetraphenylphosphonium chloride readily yielded the corresponding organic adducts, and with mono- and di-halogenated alkanes generated a series of the corresponding esters of ferrocenylphosphonofluoridodithioates. Similarly, using 1,3-epithionaphtho[1,8-*cd*][1,2,6]oxadiphosphinine 1,3-disulfide or Belleau’s Reagent in place of **FcLR** resulted in the corresponding novel salts, adducts, and ester derivatives. All new compounds have been characterized by means of multi-NMR (^1^H, ^13^C, ^31^P, ^19^F) spectroscopy and accurate mass measurement in conjunction with single crystal X-ray crystallography of four structures.

## 1. Introduction

Organophosphorus-fluorine heteroatom compounds (OPFHACs) bearing a P–F bond are of interest due to their diverse chemical or biological activities, such as selective phosphorylating agents in synthesis, and efficient inhibition of several classes of enzyme [1,2,3,4,5,6,7,8,9,10,11,12]. This new class of thiophosphates bearing one or more anion FPS**^−^** units was first prepared from the reaction of alkali metal fluorides and P_4_S_10_ by Roesky’s group [13,14]. Since then, the synthesis of thiophosphoryl halides S=PF_3_ and S=PFCl_2_, and their derivatives S=PF_2_NH_2_, S=PFClNH_2_, S=PF_2_N=PF_2_X (X = Br, NH_2_ or OH), S=PF_2_N=PCl_3_, and S=PF_2_N=PF_2_N=C=NSiMe_3_ was reported successively [15,16,17,18,19]. There are few examples of the synthesis of simple phosphonofluorodithioates ROP(S)(S^−^)F containing a fluorine atom attached directly to the phosphorus atom [15,20,21]. The nucleoside phosphonofluoridodithioate monoesters were also prepared via oxidation of nucleoside phosphonodithioate with I_2_ in pyridine in the presence of TMSCl, followed by addition of triethylamine trihydrofluoride (TAF) [22,23]. Similar analogues were obtained from a one-pot sequential reaction of 1,3,2-dithiaphospholane P(III) derivatives, which were converted readily into the corresponding P(V) compounds by addition of elemental sulfur and finally into phosphonofluoridodithioates by further treating with TBAF [24]. The importance of phosphoro-fluorine compounds in pure and applied chemistry invigorated our interest in synthesizing new phosphorodithioates bearing the P–F group. Recently, we have reported the synthesis of a series of phenylphosphonofluorodiselenoic salts, adducts, and esters [25]. Herein, we extend this procedure for the synthesis of potassium and tetrabutylammonium salts of ferrocenyl- and aryl-phosphonofluoridodithioates, and the related organic adducts and esters. To the best of our knowledge, this is the first reported synthesis and characterization of ferrocenyl-phosphonofluorodithioates [FcPS_2_F]^−^ and their structural analogues, providing a valuable addition to the library of phosphodithioate compounds.

## 2. Results and Discussion

**Scheme 1:**
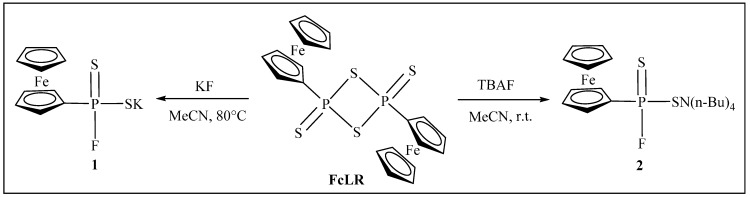
Salts **1** and **2** derived from **FcLR** and KI or TBAF.

The preparation, spectroscopic characterization, and crystal structures of a ferrocene analogue of Lawesson Reagent, 2,4-diferrocenyl-1,3,2,4-diathiadiphosphetane 2,4-disulfide {[FcP(μ-S)S]_2_, **FcLR**} has been reported by our group [26,27]. **FcLR** reacted with two equivalents of fresh dry potassium fluoride in dry acetonitrile at 80 °C under N_2_ atmosphere for 1 h, giving rise to potassium ferrocenylphosphonofluoridodithioate **1** in 97% yield; or with two equivalents of tetrabutylammonium fluoride (TBAF) in tetrahydrofuran at room temperature for 1 h providing tetrabutylammonium ferrocenylphosphonofluoridodithioate **2** in 99% yield (Scheme 1). Both reactions were fast and very straightforward and must be performed in a moisture and oxygen-free atmosphere. Treatment of **FcLR** with dry KCl or KBr in acetonitrile or with HCl and HBr in the presence of triethylamine in dry acetonitrile or tetrahydrofuran at room temperature did not result in the similar chloride and bromide products to **1** and **2**, indicating that the Cl^−^ and Br^−^ anions are much less reactive nucleophiles than the F^−^ anion.

Using an analogous process to Yilmaz and coworkers [28], salt **1** was obtained as a yellow solid and is insoluble in organic solvents but soluble in oxygen-free water and slowly decomposed when stored at room temperature. Organic salt **2** was prepared as golden sticky oil, is soluble in organic solvents, and shows good air stability at room temperature. Both compounds show the anticipated molecular ion peaks [M − K]^−^ or [M – N(*n*-Bu)_4_]^−^ and satisfactory accurate mass measurement. The ^31^P-NMR spectra exhibits doublets at δ_p_ = 132.1 ppm in compound **1** and 127.3 ppm in compound **2**, respectively, attributable to the presence of the P–F single bond, and the values are significantly bigger than that in their selenium counterpart PSe_2_ ions [25]. In the ^19^F-NMR spectra, doublets are observed with ^1^*J*(P,F) coupling constants of 1008 Hz for both **1** and **2** within the known literature values [14,25,28,29]. It should be noted that the ^1^H-NMR spectrum for compound **1** is poor quality (Figure S1); this may be explained by the inherent “shielding” effect of salt ions in the solution leading to sample conductivity [30] or, more likely, the presence of traces of oxidized paramagnetic ferrocenium species. However, the ^1^H-NMR spectrum still clearly shows that only ferrocenyl ring protons are present.

**Scheme 2:**
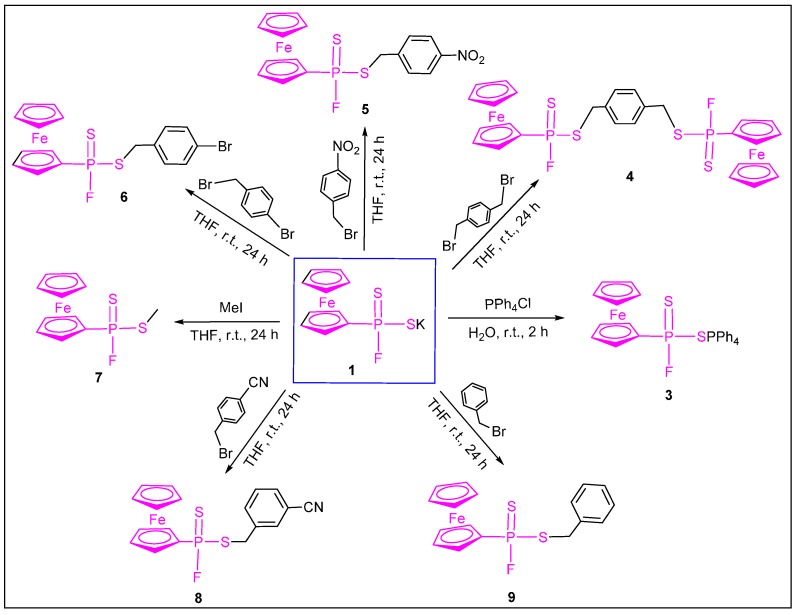
Synthesis of heteroatom derivatives **3**–**9** from salt **1**.

We presumed that compounds **1** and **2** are, like phenylphosphonofluorodiselenoic salts, strong nucleophiles [25] and therefore are able to serve as useful precursors for the synthesis of a wide variety of functionalized heteroatom systems and ligands. Compounds **1** and **2** should have the same reactivity toward organic substituents; therefore we chose compound **1** as a target staring material to explore their reactivity. Treating **1** with an equal molar amount of tetraphenylphosphonium chloride in degassed water at room temperature led to the formation of tetraphenylphosphonium ferrocenylphosphonofluoridodithioate **3** in 91% yield, though it should be noted that we have not established quantitative exchange of the cations in this or subsequent reactions. Reacting **1** with half equimolar amount of *p*-xylylene dibromide in dry tetrahydrofuran in room temperature generated 1,4-phenylenebis(methylene) bis(ferrocenylphosphonofluoridodithioate) **4** in 87% yield. Similarly, compound **1** was allowed to react with an equivalent of mono-halogenated alkanes, giving a series of esters of ferrocenylphosphonofluoridodithioates **5**–**9** in 64%–86% yields, respectively (Scheme 2). It is worth noting that products **5** and **8**, in which the phenyl groups bear the strong electron-withdrawing group NO_2_ and C≡N group, were obtained in rather lower yields (64% and 66%, respectively); thus, the results indicate that strong electron-withdrawing groups may be unfavorable.

Compounds **3**–**9** are air- and moisture-stable oils, pastes, or solids and are soluble in common organic solvents such as dichloromethane, chloroform, acetone, and tetrahydrofuran. All new compounds show the anticipated molecular ion peaks [M]^+^, and were confirmed by satisfactory accurate mass measurements. Not surprisingly, the ^31^P- and ^19^F-NMR spectra of **3** show similar patterns to **1** and **2** with identical ^1^*J*(P,F) coupling constants apart from another singlet signal in the ^31^P-NMR spectrum at δ_P_ = 23.2 ppm, assigned to the PPh_4_ cation ion. The ^31^P-NMR spectra of **4**–**9** display signals ranging from δ_P_ = 116.0 to 118.0 ppm with ^31^P–^19^F coupling constants differing from *J*(P,F) = 1098–1101 Hz. In the ^19^F-NMR spectra of compounds **4**–**9**, two equal signals in the range δ_F_ = −42.6–−38.3 ppm being considerately bigger than that in their selenium counterpart esters (δ_F_ = −58.5–−55.8 ppm) [25] are observed with the matching ^31^P–^19^F coupling constants.

**Scheme 3:**
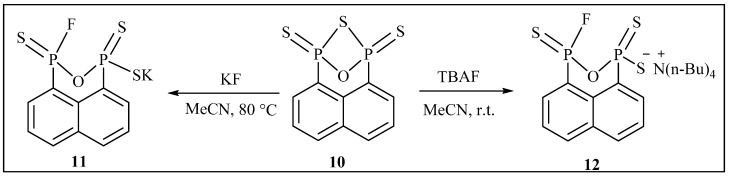
Synthesis of salts **11** and **12**.

By using the same procedure, we have carried out the synthesis of potassium 3-fluoronaphtho[1,8-*cd*][1,2,6]oxadiphosphinine-1-thiolate 1,3-disulfide 11 and tetrabutylammonium, 3-fluoronaphtho[1,8-*cd*][1,2,6]oxadiphosphinine-1-thiolate 1,3-disulfide salt **12** as shown in Scheme 3. 1,3-Epithionaphtho[1,8-*cd*][1,2,6]oxadiphosphinine 1,3-disulfide **10** was obtained from the half-oxidization of the know compound 1,3-epithionaphtho[1,8-*cd*][1,2,6]thiadiphosphinine 1,3-disulfide [31]. Treating **10** with two equivalents of fresh dry potassium fluoride at 80 °C in dry acetonitrile under N_2_ atmosphere for 1 h led to potassium 3-fluoronaphtho[1,8-*cd*][1,2,6]oxadiphosphinine-1-thiolate 1,3-disulfide **11** in 98% yield as a pale yellow solid; or with two equivalents of tetrabutylammonium fluoride (TBAF) at room temperature in tetrahydrofuran for 1 h gave tetrabutylammonium 3-fluoronaphtho[1,8-*cd*][1,2,6]oxadiphosphinine-1(3*H*)-thiolate 1,3-disulfide **12** in 85% yield as a brown sticky paste. The P–O–P bond could not be broken even when an excess of potassium fluoride or tetrabutylammonium fluoride was used, indicating that the P–O–P bridge is more robust than the P–S–P bridge in the reaction.

**Scheme 4:**
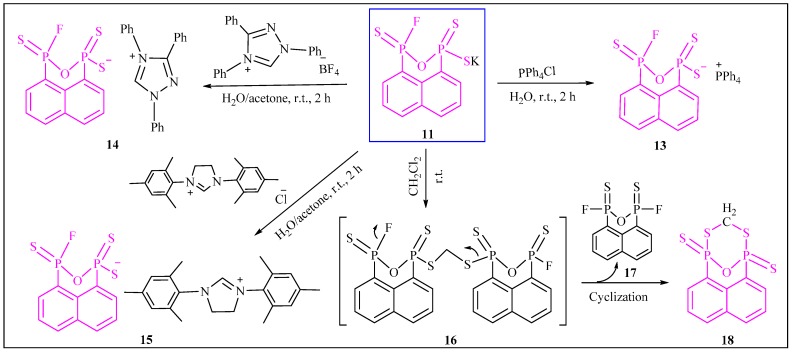
Synthesis of derivatives **13**–**18** from salt **11**.

Once again, presuming that both compounds **11** and **12** possessing the same ArP_2_OS_3_F^−^ ion should have similar reactivity, we selected salt **11** as a target starting material to explore their reactivity toward organic substituents. Complete conversion of **11** to the corresponding organic tetraphenylphosphonium salt **13** was carried on straightforwardly. Similarly, the reactions of 1,3,4-triphenyl-*1H*-1,2,4-triazol-4-ium tetrafluoroborate and 1,3-dimesityl-4,5-dihydro-*1H*-imidazol-3-ium chloride with **1** under identical conditions afforded the corresponding organic salts **14** and **15** in excellent yields (Scheme 4). Alkylation product **18** was obtained in 46% yield when **11** or **12** was dissolved in medium dichloromethane and the solution was stirred at room temperature for 48 h. It is believed that the mechanism for this reaction involved the intermediate **16**, a product of the alkylation of two molecules of **11** or **12** with one molecule of dichloromethane; afterward, the intermediate, **16**, broke and cyclized to give newly formed six-membered P_2_S_2_CO heterocyclic compound **18** by loss of a molecule of 1,3-difluoronaphtho[1,8-*cd*][1,2,6]oxadiphosphinine 1,3-disulfide **17**. Unfortunately, we are not able to grasp and identify compound **17** due possibly to its instability on the chromatography column or ready decomposition when exposed to air. Attempts to study the mechanism of the reaction by ^31^P-NMR were unsuccessful because complex mixtures were observed.

Salt **11** was obtained as a pale yellow solid and is poorly soluble in organic solvents but soluble in oxygen-free water. All of **11**–**15** are very stable under an inert atmosphere of nitrogen, but they appear to undergo slow decomposition when exposed to the air after days at room temperature. Organic salts 1**2**–**15** were synthesized as a sticky paste, foam, or solid, are soluble in organic solvents, and also show good air stability at room temperature. Heterocycle **18** was isolated as yellow foam and was very stable in air and moisture. Surprisingly, in contrast to its starting material **10**, compound **18** is soluble in normal organic solvents such as dichloromethane, chloroform, acetone, and so on. All salts show the anticipated molecular cation and anion ion peaks and satisfactory accurate mass measurement. Meanwhile, compound **18** displays the anticipated molecular ion peak and satisfactory accurate mass measurement. The ^31^P-NMR spectra of salts **11**–**15** exhibit a similar pattern of two unequal phosphorus signals ranging from δ_P_ = 106.4–107.1 ppm for the non-fluorinated phosphorus and 78.1–78.6 ppm for the fluorinated phosphorus atom with the corresponding ^1^*J*(P,F) and ^2^*J*(P,P) coupling constants. The ^1^*J*(P,F) coupling constants differing from 1099 to 1106 Hz are considerately lower than that in the analogous tetrabutylammonium, 3-fluoronaphtho[1,8-*cd*][1,2,6]thiadiphosphinine-1-thiolate 1,3-disulfide salt (1144 Hz) [32], and in the selenium counterpart PhPSe_2_F ions (1141–1146 Hz) [26]. The most significant difference is that the much bigger ^2^*J*(P,P) coupling constant of 54.0 Hz was found in **11**–**15**, comparing to its reported analogy (12.6 Hz) [33], indicating the noticeably differing effects of the P–S–P and P–O–P bridges. The singlet due to the PPh_4_ cation in **13** is observed at δ_P_ = 23.8 ppm. In the ^19^F-NMR spectra, doublets at δ_F_ = −42.6–−28.4 ppm are observed along with the matching ^1^*J*(P,F) coupling constants, falling within the literature values [14,25,28,29]. Detailed analysis revealed that the small ^3^*J*(P,F) = 3.2 or 3.3 Hz was observed in compounds **12**, **13**, and **15**. For **18**, the ^31^P-NMR spectrum shows a singlet at δ_P_ = 106.0 ppm with a ^2^*J*(P,P) coupling constant of 54.0 ppm. The ^1^H-NMR spectrum displays signals from the aryl and CH_2_ protons present within compound. 

**Figure 1:**
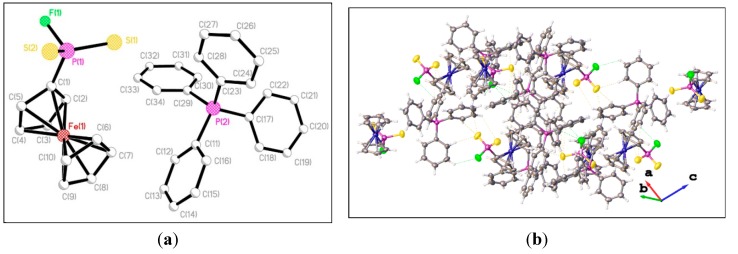
(**a**) Single crystal structure of **3** (Hydrogen atoms omitted for clarity). Selected bond lengths (Å) and angles (°) (esds in parentheses): S(1)–P(1) 1.963(3), S(2)–P(1) 1.953(3), P(1)–F(1), 1.615(4), P(1)–C(1) 1.801(6), P(1)∙∙∙P(2) 7.096; S(1)–P(1)–F(1) 105.70(18), S(2)–P(1)–F(1) 106.35(19), F(1)–P(1)–C(1) 98.6(2), S(1)–P(1)–S(2) 121.66(11), S(1)–P(1)–C(1) 110.7(2), S(2)–P(1)–C(1) 111.0(2); (**b**) Packing diagram of **3**.

**Figure 2:**
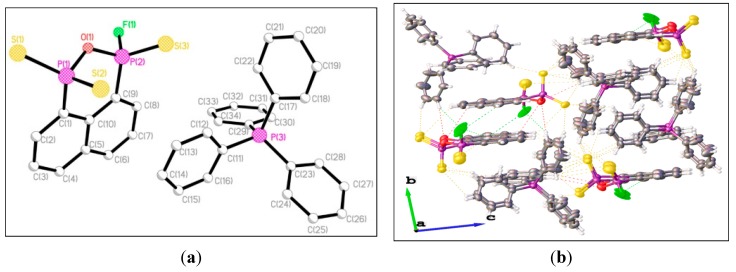
(**a**) Single crystal structure of **13** (hydrogen atoms omitted for clarity). Selected bond lengths (Å) and angles (°) (esds in parentheses): S(1)–P(1) 1.957(3), S(2)–P(1) 1.957(3), S(3)–P(2) 1.883(4), P(1)–O(1) 1.689(6), P(1)–C(1) 1.813(8), P(2)–F(1) 1.593(8), P(2)–O(1) 1.573(6), P(2)–C(9) 1.794(8); P(1)–O(1)–P(2) 125.5(4), S(1)–P(1)–S(2) 121.42(13), S(1)–P(1)–O(1) 105.0(2), S(1)–P(1)–C(1) 111.9(3), S(2)–P(1)–O(1) 107.9(2), S(2)–P(1)–C(1) 108.6(3), O(1)–P(1)–C(1) 99.8(3), S(3)–P(2)–F(1) 100.7(4), S(3)–P(2)–O(1) 119.5(3), S(3)–P(2)–C(9) 120.0(3), F(1)–P(2)–O(1) 102.4(4), F(1)–P(2)–C(9) 107.3(4), O(1)–P(2)–C(9) 104.9(4); (**b**) Packing diagram of **13**.

**Figure 3:**
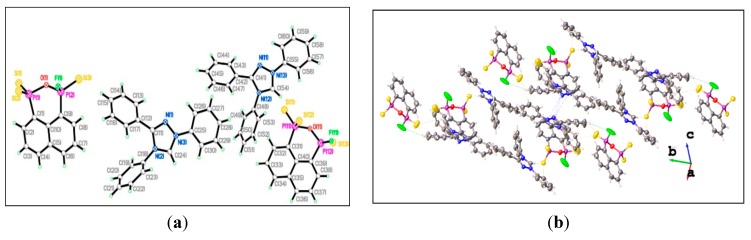
(**a**) Single crystal structure of **14**. Selected bond lengths (Å) and angles (°) (esds in parentheses) (dimensions for second independent molecule in square parentheses): S(1)–P(1) 1.950(4) [1.955(5)], S(2)–P(1) 1.957(4) [1.974(4)], S(3)–P(2) 1.891(4) [1.819(5)], P(1)–O(1) 1.680(6) [1.671(6)], P(1)–C(1) 1.816(10) [1.817(9)], P(2)–F(1) 1.563(8) [1.715(8)], P(2)–O(1) 1.577(8) [1.569(8)], P(2)–C(9) 1.776(10) [1.772(9)]; S(1)–P(1)–S(2) 117.45(17) [117.88(17)], S(1)–P(1)–O(1) 105.7(3) [105.6(3)], S(1)–P(1)–C(1) 112.7(4) [110.9(4)], S(2)–P(1)–O(1) 108.7(3) [110.3(3)], S(2)–P(1)–C(1) 111.7(4) [111.6(3)], O(1)–P(1)–C(1) 98.6(4) [98.6(4)], S(3)–P(2)–F(1) 103.8(4) [103.8(4)], S(3)–P(2)–O(1) 114.3(3) [113.5(3)], S(3)–P(2)–C(9) 118.7(4) [113.0(4)], F(1)–P(2)–O(1) 106.7(5) [109.1(5)], F(1)–P(2)–C(9) 106.3(6) [111.7(5)], O(1)–P(2)–C(9) 106.2(5) [105.8(5)], P(1)–O(1)–P(2) 128.0(4) [128.9(4)]; (**b**) Packing diagram of **14**.

Crystals of **3**, **13**, **14**, and **18** suitable for X-ray analysis were obtained by diffusion of hexane into the dichloromethane solution at room temperature. Crystal data and structure refinement are summarized in Table 1. All structures have a single molecule of the compound in the asymmetric unit, aside from compound **14**, in which the asymmetric unit comprises two independent molecules. The X-ray structural analysis of **3**, **13**, and 1**4** as shown in Figure 1, Figure 2 and Figure 3 reveals they crystallize as cation and anion ion-separated species. The X-ray structure of **3** shows that the ferrocenylphosphonofluoridodithioate anionic part contains a distorted tetrahedral phosphorus atom with P–S bond distances of 1.963(3) and 1.953(3) Å, which are shorter than in the reported ferrocenylphosphonodithioate [Fc(RO)PS_2_]^−^ complexes [1.973(2)–2.046(2) Å] [33,34]; however, the values are still intermediate between the P–S single bond [*ca*. 2.10 Å] [35] and the P=S double bond [*ca*. 1.91 Å] [36], indicating the delocalization of the negative charge over the FPS_2_^−^ fragment. The P–F bond length [1.615(4) Å] is statistically indistinguishable from that in PSe_2_F^−^ [1.604(3)–1.610(5) Å] [25]. The three-dimensional network shows the weak intramolecular and intermolecular C–H···S interactions (yellow dashed line) and intermolecular C–H···F interactions (blue dashed line) forming the polymeric architecture in **3** as shown in Figure 1b.

**Table 1:** Details of the X-ray data collections and refinements for **3**, **13**, **14**, and **18**.

Compound	3	13	14	18
Formula	C_34_H_29_FFeP_2_S_2_	C_34_H_26_FOP_3_S_3_	C_30_H_22_FN_3_OP_3_S_3_	C_10_H_6_O_2_P_2_S_4_
M	638.52	658.68	617.65	348.34
Crystal system	Monoclinic	Monoclinic	Triclinic	Monoclinic
Space group	P2_1_/n	P2_1_/c	P-1	P2_1_/n
a/Å	11.704(2)	9.563(3)	9.992(4)	11.171(3)
b/Å	17.994(2)	16.876(5)	16.808(8)	8.618(2)
c/Å	14.981(3)	19.258(6)	18.117(8)	14.122(4)
α	90	90	104.020(11)	90
β	106.902(9)	93.820(7)	90.270(10)	99.002(6)
γ	90	90	104.404(11)	90
U/A^3^	3018.6(9)	3101.1(15)	2852(3)	1342.8(6)
*Z*	4	4	4	4
µ/mm^−1^	7.726	4.277	4.090	9.32
Reflections collected	25,726	23,431	21,800	9765
Independent reflections	5310	5430	9960	2346
*R*_int_	0.1707	0.1124	0.1052	0.0696
R1	0.0824	0.1037	0.0988	0.0526
wR2 [I > 2σ(I)]	0.0912	0.2408	0.2433	0.1339

The X-ray structure of **13** reveals that the naphthalene part of the molecule and two phosphorus atoms lie very close to the mean plane fitted to these atoms [maximum deviation 0.092 Å for C(1)], with the oxygen atom lying 0.548 Å above the mean plane and its cationic part being identical to that in **3**. The structures of the cation in compounds **3** and **13** are noteworthy. The orientations of four phenyl ring are distorted away from each other, as evidenced by the different dihedral angles between three facing anion ion phenyl rings and one away phenyl ring (78.64°, 71.03° and 57.38° in **3** and 68.41°, 75.63° and 39.73° in **13**) in the cation Ph_4_P^+^ ions. This distortion presumably arises as a result of steric interactions of the Ph_4_P^+^ cation ion with the anionic fragments FPS_2_^−^ and C_10_H_6_P_2_S_3_OF^−^. The three-dimensional network in Figure 2b shows the weak intramolecular and intermolecular C–H···S interactions (yellow dashed line), intermolecular C–H···F interactions (blue dashed line), intermolecular C–H···O interactions (pink dashed line), and π–π stacking interactions responsible for the stabilization of the crossed-layers supramolecular assembly.

The single crystal structure of compound **14** has the same anionic part as that in the structure of **13**. However, in contrast to **13**, the C_10_H_6_P_2_ part of the anion is planar with the oxygen atom being significantly distorted and lying 0.495 [0.490] Å above the mean plane. The C_3_P_2_O ring is buckled with the C_3_P_2_ and P_2_O planes, inclined with respect to each other by 46.3° in **13** and 42.0° [44.1°] in **14**. It is worth noting that the opening of the P_2_SO ring results in substantial lengthening of the transanular P···P distances {2.900 Å in **13** and 2.923 Å [2.927 Å] in **14** *vs.* 2.73 Å} [35,37]. The internal P–O–P angle in **13** [125.5(4)°] is significantly smaller than the corresponding values in **14** {128.0(4) [128.9(4)]°}, indicating the steric effect of the different cationic parts. However, the P–O–P angles are different from each other {125.5(4)° in **13** and 128.0(4) [128.9(4)]° in **14**}; these values are much wider than in similar P–S–P structures [100.21(4)–102.69(4)°] [31,35,37]. Surprisingly, the two P–F bond distances in **14** [1.563(8) Å] are slightly shorter than in **13** [1.593(8) Å], **2** [1.615(4) Å], and PSe**2**F^−^ [1.604(3)–1.610(5) Å] [25]. All P–S bond lengths are not substantially different from those [1.950(4)–1.974(4) Å] in **2**, **13**, and **14**, falling between the P–S single bond lengths [2.3307(8) Å] and the P=S double bond lengths [1.8939(12) Å] [35,38]. The P=S distances in **13** [1.883(4) Å] and **14** {1.891(4) [1.819(5)] Å} are slightly shorter than that in PO_3_S systems [1.888(8)–1.8987(16) Å] [38], and two different P–O bond lengths are observed in **13** or **14**, in which one P–O bond distance {1.689(6) or 1.680(6) [1.671(6)] Å} is significantly longer than the other {1.573(6) or 1.577(8) [1.569(8)] Å}. The aggregation in Figure 3b is dominated by the weak intramolecular and intermolecular C–H···S interactions (yellow dashed line), intermolecular C–H···F interactions (blue dashed line), intermolecular C–H···O interactions (pink dashed line), which involve the H atoms from the aryl rings and the triazole rings, to construct the crossed-layers supramolecular assembly without the presence of the π–π stacking interactions.

**Figure 4:**
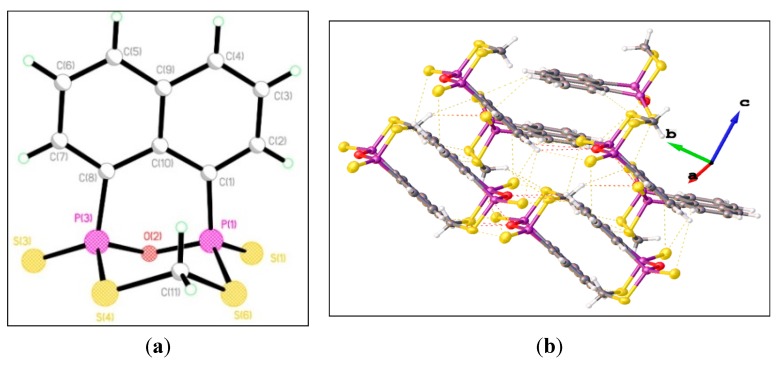
(**a**) Singlecrystal structure of **18**. Selected bond lengths (Å) and angles (°) (esds in parentheses): P(1)–S(1) 1.9020(13), P(3)–S(3) 1.9097(13), P(1)–S(6) 2.0762(14), P(3)–S(4) 2.0730(14), P(1)–O(2) 1.629(2), P(3)–O(2) 1.628(2), S(4)–C(11) 1.830(4), S(6)–C(11) 1.828(4); S(1)–P(1)–S(6) 111.30(6), S(1)–P(1)–C(1) 117.56(11), S(1)–P(1)–O(2) 113.27(9), S(6)–P(1)–C(1) 107.31(12), S(6)–P(1)–O(2) 103.96(9), P(1)–S(6)–C(11) 99.25(12), P(3)–S(4)–C(11) 100.33(12), O(2)–P(1)–C(1) 102.22(13), S(3)–P(3)–S(4) 110.09(6), S(3)–P(3)–O(2) 112.10(9), S(3)–P(3)–C(8) 118.41(11), S(4)–P(3)–O(2) 105.51(9), S(4)–P(3)–C(8) 107.80(12), O(2)–P(3)–C(8) 101.96(13), P(1)–O(2)–P(3) 127.10(14); (**b**) Packing structure of **18**.

The X-ray structure of **18** as shown in Figure 4 reveals a very different geometry, compared to the structures of **2**, **13** and **14**. The naphthalene part of the molecule and two phosphorus atoms lies very close to a mean plane [maximum deviation 0.071 Å for P(3)]. The C_3_P_2_O is non-planar with a dihedral angle of 46.73° about P···P axis. The small newly formed six-membered P_2_S_2_CO ring is notable distorted from planar: atom O(2) lies 0.631 Å below and atom C(11) lies 0.915 Å above the P(1)–P(3)–S(4)–S(6) mean plane with two S(1) and S(3) atoms below the mean plane. The newly formed eight-membered C_3_P_2_S_2_C ring is hinged with the C_3_P_2_ and P_2_S_2_ mean planes inclined by 74.2° with respect to each other. The transanular P···P distance is 2.916 Å. The P(1)–S(1) [1.9020(13) Å] and P(3)–S(3) [1.9097(13) Å] and P(1)–S(6) [2.0762(14) Å] and P(3)–S(4) [2.0730(14) Å] distances are comparable to those in other known C_3_P_2_S ring derivatives [31,36,37,39]. The supramolecular structure of **18** consists of a combination of weak intermolecular C–H···S interactions (yellow dashed line), intermolecular C–H···O interactions (pink dashed line), and π–π stacking interactions to build up the multi-stepped supramolecular assembly shown in Figure 4b.

**Scheme 5:**
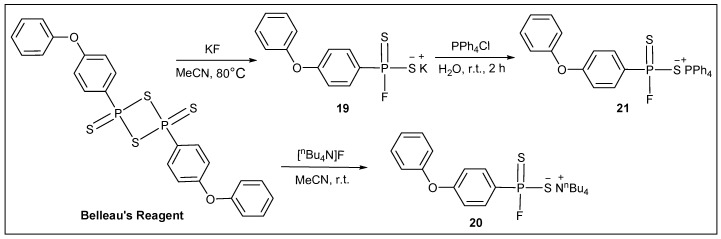
Synthesis of derivatives **19**–**21** from Belleau’s Reagent.

Using the Yilmaz methodology [28], we also carried out a similar reaction with Belleau’s Reagent. The reaction of Belleau’s Reagent with two molar equivalents of dry potassium fluoride at 80 °C gave potassium (4-phenoxyphenyl)phosphonofluoridodithioate **19** in 97% yield as a pale yellow paste; or with two equivalents of tetrabutylammonium fluoride (TBAF) at room temperature in tetrahydrofuran for 1 h it led to tetrabutylammonium (4-phenoxyphenyl)phosphonofluoridodithioate **20** in 99% yield as a slightly greenish yellow oil (Scheme 5). Furthermore, reacting **19** with equivalent of tetraphenylphosphonium chloride [28] at room temperature in degassed water/acetone medium furnished tetraphenylphosphonium (4-phenoxyphenyl)phosphonofluoridodithioate **21** in 86% yield as a white foam. All three reactions took place immediately and must be performed in a moisture- and oxygen-free atmosphere. Similar to salts **1** and **11**, compound **19** is poorly soluble in organic solvents but soluble in oxygen-free water and is air and moisture stable. Compounds **20** and **21** are insoluble in oxygen-free water but soluble in normal organic solvents. The ^31^P-NMR spectra of salts **19**–**21** reveal similar pattern to salts **1**–**3**. Doublets at δ_P_ = 130.2, 126.3, and 126.4 ppm, respectively, were found with the ^1^*J*(P,F) coupling constants differing from 1016 to 1028 Hz in **19**–**21**. The ^19^F-NMR spectra of **19**–**21** displays doublets at δ_F_ = −26.3, −20.9, and −35.3 ppm, quite similar to literature values [14,25,28,29].

## 3. Experimental Section

### 3.1. General

Unless otherwise stated, all reactions were carried out under on oxygen-free nitrogen atmosphere using pre-dried solvents and standard Schlenk techniques; subsequent chromatographic and work-up procedures were performed in air. NMR spectra were recorded on Bruker Avance-400 (^1^H at 400 MHz, ^13^C at 100.6 MHz, ^31^P at 162.0 MHz, and ^19^F at 376.5 MHz, Blue Lion Biotech, Carnation, WA, USA) and JEOL GSX-270 (^77^Se at 51.5 MHz referenced to external Me_2_Se, JEOL USA, Inc. Peabody, MA, USA) at 25 °C (unless stated otherwise). IR spectra were recorded as KBr pellets in the range of 4000–250 cm^−1^ on a Perkin-Elmer 2000 FTIR/Raman spectrometer (Perkin Elmer, Beaconsfield, UK). Crystals of **3**, **13**, **14**, and **18** suitable for X-ray analysis were obtained by diffusion of hexane into the dichloromethane solution at room temperature. X-ray crystal structures were determined at −148(1) °C on a Rigaku ACTOR-SM, Saturn 724 CCD area detector [the St Andrews Automated Robotic Diffractometer (STANDARD), Rigaku, Houston, TX, USA] [40] with SHINE optic using Mo Ka radiation (k = 0.71073 Å). The data were corrected for Lorentz, polarization, and absorption. The data was collected and processed using CrystalClear (Rigaku) [41]. The structures were solved by direct methods [42] and expanded using Fourier techniques [43]. Hydrogen atoms were refined using the riding model. All calculations were performed using the CrystalStructure [44] and SHELXL 97 [45]. CCDC contains the supplementary crystallographic data for this paper. These data can be obtained free of charge via www.ccdc.cam.ac.uk/conts/retrieving.html or from the Cambridge Crystallographic Data Center, 12 Union Road, Cambridge CB2 1EZ, UK; Fax (+44)-1223-336-033; E-Mail: deposit@ccdc.cam.ac.uk. CCDC No. 1061138-1061141.

### 3.2. Synthesis

#### 3.2.1. Synthesis of Potassium Ferrocenylphosphonofluoridodithioate (**1**)

A mixture of 2,4-diferrocenyl-1,3,2,4-diathiadiphosphetane 2,4-disulfide (**FcLR**) (1.12 g, 2.0 mmol) and freshly dried and finely ground KF (0.232 g, 4.0 mmol) in dry acetonitrile (50 mL) was stirred at ambient temperature. After mixing for 10 min, the mixture was heated at 80 °C for 1 h. Upon cooling to room temperature the reaction mixture was filtered and the filtrate was evaporated *in vacuo* to give the title compound **1** as yellow solid (1.305 g, 97%). M.p. 147–149 °C. Selected IR (KBr, cm^−1^): 1638 (w), 1411 (m), 1259 (m), 1176 (m), 1106 (m), 1019(s), 812(m), 760(s), 697(vs), 598(s), 502 (s), 435 (m). ^1^H-NMR (D_2_O, δ), 4.65–4.48 (m, 4H, ferrocenyl-H), 4.36 (s, 5H, ferrocenyl-H) ppm. ^13^C-NMR (D_2_O, δ), 71.7 (d, *J*(P,C) = 13.1 Hz, ferrocenyl-C), 71.5 (d, *J*(P,C) = 20.1 Hz, ferrocenyl-C), 71.1 (d and weak, *J*(P,C) = 57.9 Hz, ferrocenyl-C), 70.6 (s, ferrocenyl-C) ppm. ^31^P-NMR (D_2_O, δ), 132.1 (d, *J*(P,F) = 1016 Hz) pm. ^19^F-NMR (282.34 MHz, D_2_O, δ), −18.7 (d, *J*(F,P) = 1016 Hz) ppm. MS (CI^−^, *m*/*z*), 299 [C_10_H_9_FFePS_2_]^−^. Accurate mass measurement (CI^−^MS): 298.9208 [C_10_H_9_FFePS_2_]^−^, calculated mass for [C_10_H_9_FFePS_2_]^−^: 298.9222.

#### 3.2.2. Synthesis of Tetrabutylammonium Ferrocenylphosphonofluoridodithioate (**2**)

A suspension of **FcLR** (3.36 g, 6.0 mmol) and tetrabutylammonium fluoride (12 mL of 1 M solution in THF, 12.0 mmol) in THF (50 mL) was heated at 80 °C for 1 h. Upon cooling to room temperature the reaction mixture was filtered to remove unreacted solid and the filtrate was evaporated *in vacuo* to give the title compound **2** as golden sticky oil (6.470 g, 99%). Selected IR (KBr, cm^−1^): 1638 (m), 1467 (s), 1382 (m), 1172 (m), 1106 (m), 1023 (s), 884 (m), 822 (m), 738 (m), 698 (vs), 586 (m), 489 (s). ^1^H-NMR (CD_2_Cl_2_, δ), 4.60–4.55 (m, 2H, ferrocenyl-H), 4.31–4.27 (m, 2H, ferrocenyl-H), 4.22 (s, 5H, ferrocenyl-H), 3.25–3.18 (m, 8H, CH_2_), 1.67–1.58 (m, 8H, CH_2_), 1.42–1.38 (m, 8H, CH_2_), 0.97 (t, *J*(H,H) = 6.6 Hz, 12H, CH_3_) ppm. ^13^C-NMR (CD_2_Cl_2_, δ), 72.5 (d, *J*(P,C) = 67.6 Hz, ferrocenyl-C), 71.5 (d, *J*(P,C) = 15.5 Hz, ferrocenyl-C), 70.6 (d, *J*(P,C) = 17.7 Hz, ferrocenyl-C), 70.2 (s, ferrocenyl-C), 59.0, 24.1, 19.9, 13.6 ppm. ^31^P-NMR (CD_2_Cl_2_, δ), 127.3 (d, *J*(P,F) = 1009 Hz) pm. ^19^F-NMR (CD_2_Cl_2_, δ), −14.0 (d, *J*(F,P) = 1008 Hz) ppm. MS (CI^+^, *m*/*z*), 242 [C_16_H_36_N]^+^. Accurate mass measurement (CI^+^MS): 242.2840 [C_16_H_36_N]^+^, calculated mass for [C_16_H_36_N]^+^: 242.2846; MS (CI^−^, *m*/*z*), 299 [C_10_H_9_FFePS_2_]^−^. Accurate mass measurement (CI^−^MS): 298.9260 [C_10_H_9_FFePS_2_]^−^, calculated mass for [C_10_H_9_FFePS_2_]^−^: 298.9269.

#### 3.2.3. Synthesis of Tetraphenylphosphonium Ferrocenylphosphonofluoridodithioate (**3**)

Potassium ferrocenylphosphonofluoridodithioate (0.339 g, 1.0 mmol) was dissolved in degassed water (10 mL). To this solution was added dropwise an equivalent of tetraphenylphosphonium chloride (0.374 g, 1.0 mmol) in water (10 mL). The precipitation was almost immediately formed and the mixture was allowed to continue stirring for 1 h. The salt was harvested by filtration and was dried over 110 °C *in vacuo* to give the title compound **3** as pale yellow solid (0.581 g, 91%). M.p. 175–176 °C. Selected IR (KBr, cm^−1^): 1585 (m), 1483 (m), 1437 (s), 1168 (m), 1107 (s), 1021 (m), 997 (m), 817 (m), 758 (m), 721 (s), 690 (vs), 585 (m), 529 (s). ^1^H-NMR (CD_2_Cl_2_, δ), 7.97–7.91 (m, 4H, Ar-H), 7.81–7.71 (m, 8H, Ar-H), 7.67–7.63 (m, 8H, Ar-H), 4.60–4.57 (m, 2H, ferrocenyl-H), 4.24 (s, 5H, ferrocenyl-H), 4.22–4.20 (m, 2H, ferrocenyl-H) ppm. ^13^C-NMR (CD_2_Cl_2_, δ), 136.6 (d, *J*(P,C) = 3.0 Hz, Ar-C), 135.3 (d, *J*(P,C) = 10.1 Hz, Ar-C), 131.5 (d, *J*(P,C) = 12.7 Hz, Ar-C), 118.3 (d, *J*(P,C) = 89.8 Hz, Ar-C), 72.3 (d, *J*(P,C) = 15.2 Hz, ferrocenyl-C), 71.3 (d, *J*(P,C) = 56.4 Hz, ferrocenyl-C), 70.9 (s, ferrocenyl-C), 70.4 (d, *J*(P,C) = 13.1 Hz, ferrocenyl-C) ppm. ^31^P-NMR (Acetone-*d*_6_, δ), 126.8 (d, *J*(P,F) = 1010 Hz), 23.2 (s) pm. ^19^F-NMR (Acetone-*d*_6_, δ), −14.5 (d, *J*(F,P) = 1008 Hz) ppm. MS (CI^−^, *m*/*z*), 299 [C_10_H_9_FFePS_2_]^−^. Accurate mass measurement (CI^−^MS): 298.9210 [C_10_H_9_FFePS_2_]^−^, calculated mass for [C_10_H_9_FFePS_2_]^−^: 298.9222. MS (CI^+^, *m*/*z*), 339 [C_24_H_20_P]^+^. Accurate mass measurement (CIMS): 339.1299 [C_24_H_20_P]^+^, calculated mass for [C_24_H_20_P]^+^: 339.1297.

#### 3.2.4. Synthesis of 1,4-Phenylenebis(methylene) Bis(ferrocenylphosphonofluoridodithioate) (**4**)

A mixture of tetrabutylammonium ferrocenylphosphonofluoridodithioate (0.108 g, 2.0 mmol) in THF (30 mL) was added to *p*-xylene dibromide (0.264 g, 1.0 mmol). The mixture was stirred at room temperature for 24 h. After filtration to remove the unreacted solid and drying under reduced pressure, the residue was purified by column chromatography on silica gel using dichloromethane as eluent to give the title compound **4** as greenish yellow oil (0.608 g, 87%). Selected IR (KBr, cm^−1^): 1492 (w), 1454 (w), 1412 (m), 1186 (s), 1107 (m), 1027 (s), 1002 (m), 820 (s), 764 (m), 700 (s), 607 (m), 548 (s), 488 (s). ^1^H-NMR (CD_2_Cl_2_, δ), 7.36–7.27 (m, 4H, Ar-H), 4.70–4.60 (m, 8H, ferrocenyl-H), 4.57 (s, 10H, ferrocenyl-H), 4.37–4.31 (m, 4H, CH_2_) ppm. ^13^C-NMR (CD_2_Cl_2_, δ), 131.2 (Ar-C), 131.0 (Ar-C), 129.4 (Ar-C), 128.7 (Ar-C), 75.0 (d, *J*(P,C) = 75.1 Hz, ferrocenyl-C), 72.6 (d, *J*(P,C) = 13.5 Hz, ferrocenyl-C), 72.2 (d, *J*(P,C) = 19.3 Hz, ferrocenyl-C), 70.8 (s, ferrocenyl-C), 31.0 ppm. ^31^P-NMR (CD_2_Cl_2_, δ), 115.5 (d, *J*(P,F) = 1103 Hz) pm. ^19^F-NMR (CD_2_Cl_2_, δ), −39.6 (d, *J*(F,P) = 1104 Hz) ppm. MS (EI^+^, *m*/*z*), 702 [C_28_H_26_F_2_Fe_2_P_2_S_4_]^+^. Accurate mass measurement (EI^+^MS): 701.9057 [C_28_H_26_F_2_Fe_2_P_2_S_4_]^+^, calculated mass for [C_28_H_26_F_2_Fe_2_P_2_S_4_]^+^: 701.9059.

#### 3.2.5. Synthesis of 4-Nitrobenzyl Ferrocenylphosphonofluoridodithioate (**5**)

A mixture of tetrabutylammonium ferrocenylphosphonofluoridodithioate (0.541 g, 1.0 mmol) in THF (30 mL) was added to 4-nitrobenzyl bromide (0.216 g, 1.0 mmol). The mixture was stirred at room temperature for 24 h. After filtration to remove the unreacted solid and drying under reduced pressure, the residue was purified by column chromatography on silica gel using dichloromethane as eluent to give the title compound **5** as pale yellow paste (0.286 g, 64%). Selected IR (KBr, cm^−1^): 1604 (m), 1521 (s), 1347 (s), 1186 (m), 1108 (m), 1029 (m), 821 (m), 801 (m), 699 (s), 548 (m), 493 (m). ^1^H-NMR (CD_2_Cl_2_, δ), 8.17 (d, *J*(H,H) = 7.5 Hz, 2H, Ar-H), 7.56 (d, *J*(H,H) = 7.5 Hz, 2H, Ar-H), 4.64–4.34 (m, 6H, CH_2_ + ferrocenyl-H), 4.33 (s, 5H, ferrocenyl-H) ppm. ^13^C-NMR (CD_2_Cl_2_, δ), 144.9 (Ar-C), 130.0 (Ar-C), 129.4 (Ar-C), 123.8 (Ar-C), 72.7 (d, *J*(P,C) = 13.6 Hz, ferrocenyl-C), 72.2 (d, *J*(P,C) = 19.7 Hz, ferrocenyl-C), 71.1 (d, *J*(P,C) = 14.5 Hz, ferrocenyl-C), 70.8 (s, ferrocenyl-C), 36.7 (SCH_2_) ppm. ^31^P-NMR (CD_2_Cl_2_, δ), 116.0 (d, *J*(P,F) = 1103 Hz) ppm. ^19^F-NMR (CD_2_Cl_2_, δ), −38.8 (d, *J*(F,P) = 1104 Hz) ppm. MS (CI^+^, *m*/*z*), 453 [C_17_H_15_FFeNO_2_PS_2_NH_4_]^+^. Accurate mass measurement (CI^+^MS): 451.0002 [C_17_H_15_F^54^FeNO_2_PS_2_NH_4_]^+^, calculated mass for [C_17_H_15_F^54^FeNO_2_PS_2_NH_4_]^+^: 451.0000.

#### 3.2.6. Synthesis of 4-Bromobenzyl Ferrocenylphosphonofluoridodithioate (**6**)

A mixture of tetrabutylammonium ferrocenylphosphonofluoridodithioate (0.567 g, 1.05 mmol) in THF (30 mL) was added to 4-bromobenzyl bromide (0.260 g, 1.05 mmol). The mixture was stirred at room temperature for 24 h. After filtered to remove unreacted solid and dried under reduced pressure, the residue was purified by column chromatography on silica gel using dichloromethane as eluent to give the title compound **6** as reddish yellow paste (0.400 g, 86%). Selected IR (KBr, cm^−1^): 1468 (m), 1440 (m), 1412 (m), 1186 (s), 1107 (m), 1027 (s), 820 (s), 760 (m), 730 (m), 699 (vs), 548 (s), 489 (s). ^1^H-NMR (CD_2_Cl_2_, δ), 7.57 (d, 2H, *J*(H,H) = 8.0 Hz, Ar-H), 7.26 (d, 2H, *J*(H,H) = 8.0Hz, Ar-H), 4.63–4.36 (m, 6H, CH_2_ + ferrocenyl-H), 4.34 (s, 5H, ferrocenyl-H) ppm. ^13^C-NMR (CD_2_Cl_2_, δ), 133.1 (Ar-C), 131.4 (Ar-C), 129.6 (Ar-C), 127.9 (Ar-C), 72.3 (d, *J*(P,C) = 13.4 Hz, ferrocenyl-C), 72.1 (d, *J*(P,C) = 19.7 Hz, ferrocenyl-C), 71.3 (d, *J*(P,C) = 15.6 Hz, ferrocenyl-C), 70.8 (s, ferrocenyl-C), 38.1 (SCH_2_) ppm. ^31^P-NMR (CD_2_Cl_2_, δ), 116.4 (d, *J*(P,F) = 1103 Hz) ppm. ^19^F-NMR (CD_2_Cl_2_, δ), −38.3 (d, *J*(F,P) = 1105 Hz) ppm. MS (CI^+^, *m*/*z*), 469 [C_17_H_15_BrFFePS_2_H]]^+^. Accurate mass measurement (CI^+^MS): 466.8988 [C_17_H_15_BrF^54^FePS_2_H], calculated mass for [C_17_H_15_BrF^54^FePS_2_H]^+^: 466.8989.

#### 3.2.7. Synthesis of Methylyl Ferrocenylphosphonofluoridodithioate (**7**)

A mixture of tetrabutylammonium ferrocenylphosphonofluoridodithioate (0.541 g, 1.0 mmol) in THF (30 mL) was added to iodomethane (0.142 g, 1.0 mmol). The mixture was stirred at room temperature for 24 h. After filtration to remove the unreacted solid and drying under reduced pressure, the residue was purified by column chromatography on silica gel using dichloromethane as eluent to give the title compound **7** as red oil (0.270 g, 86%). Selected IR (KBr, cm^−1^): 1413 (m), 1312 (m), 1186 (s), 1107 (m), 1028 (s), 1003 (w), 820 (vs), 699 (vs), 551 (s), 492 (s). ^1^H-NMR (CD_2_Cl_2_, δ), 4.67–4.39 (m, 4H, ferrocenyl-H), 4.36 (s, 5H, ferrocenyl-H), 2.44 (dd, *J*(P,H) = 16.5 Hz, *J*(F,H) = 1.7 Hz, 3H, SCH_3_) ppm. ^13^C-NMR (CD_2_Cl_2_, δ), 72.5 (d, *J*(P,C) = 12.6 Hz, ferrocenyl-C), 72.2 (d, *J*(P,C) = 19.3 Hz, ferrocenyl-C), 70.8 (s, ferrocenyl-C), 14.6 (d, *J*(P,C) = 4.2 Hz, SCH_3_) ppm. ^31^P-NMR (CD_2_Cl_2_, δ), 118.0 (d, *J*(P,F) = 1099 Hz) pm. ^19^F-NMR (CD_2_Cl_2_, δ), −42.6 (d, *J*(F,P) = 1102 Hz) ppm. MS (CI^+^, *m*/*z*), 315 [M + H]^+^. Accurate mass measurement (CI^+^MS): 312.9569 [M + H]^+^, calculated mass for [C_11_H_12_F^54^FePS_2_H]^+^: 312.9571.

#### 3.2.8. Synthesis of 4-Cyanobenzyl Ferrocenylphosphonofluoridodithioate (**8**)

A mixture of tetrabutylammonium ferrocenylphosphonofluoridodithioate (0.541 g, 1.0 mmol) in THF (30 mL) was added to 4-cyanobenzyl bromide (0.198 g, 1.0 mmol). The mixture was stirred at room temperature for 24 h. After filtration to remove the unreacted solid and driying under reduced pressure, the residue was purified by column chromatography on silica gel using dichloromethane as eluent to give the title compound **8** as pale golden paste (0.290 g, 66%). Selected IR (KBr, cm^−1^): 2229 (s), 1484 (m), 1433 (m), 1411 (m), 1276 (m), 1186 (s), 1028 (m), 1002 (m), 905 (m), 823 (s), 804 (s), 701 (vs), 601 (m), 545 (s), 492 (s). ^1^H-NMR (CD_2_Cl_2_, δ), 7.69–7.34 (m, 4H, Ar-H), 4.60–4.35 (m, 4H, ferrocenyl-H), 4.25 (s, 5H, ferrocenyl-H), 4.28–4.22 (m, 2H, CH_2_) ppm. ^13^C-NMR (CD_2_Cl_2_, δ), 133.6 (Ar-C), 132.9 (Ar-C), 132.6 (Ar-C), 132.1 (Ar-C), 131.4 (Ar-C), 129.7 (Ar-C), 72.6 (d, *J*(P,C) = 13.6 Hz, ferrocenyl-C), 72.3 (d, *J*(P,C) = 19.6 Hz, ferrocenyl-C), 71.1 (d, *J*(P,C) = 15.4 Hz, ferrocenyl-C), 70.8 (s, ferrocenyl-C), 36.6 (SCH_2_) ppm. ^31^P-NMR (CD_2_Cl_2_, δ), 116.1 (d, *J*(P,F) = 1101 Hz), 116.0 (d, *J*(P,F) = 1101 Hz) pm. ^19^F-NMR (CD_2_Cl_2_, δ), −39.1 (d, *J*(F,P) = 1103 Hz) ppm. MS (CI^+^, *m*/*z*), 433 [C_18_H_15_FFeNPS_2_NH_4_]^+^. Accurate mass measurement (CI^+^MS): 431.0101 [C_18_H_15_F^54^FeNPS_2_NH_4_]^+^, calculated mass for [C_18_H_15_F^54^FeNPS_2_NH_4_]^+^: 431.0102.

#### 3.2.9. Synthesis of Benzyl Ferrocenylphosphonofluoridodithioate (**9**)

A mixture of tetrabutylammonium ferrocenylphosphonofluoridodithioate (0.541 g, 1.0 mmol) in THF (30 mL) was added to benzyl bromide (0.170 g, 1.0 mmol). The mixture was stirred at room temperature for 24 h. After filtration to remove the unreacted solid and drying under reduced pressure, the residue was purified by column chromatography on silica gel using dichloromethane as eluent to give the title compound **9** as reddish-yellow oil (0.312 g, 80%). Selected IR (KBr, cm^−1^): 1493 (m), 1454 (m), 1412 (m), 1186 (s), 1107 (m), 1027 (s), 1002 (m), 820 (vs), 698 (vs), 548(s), 492 (s). ^1^H-NMR (CD_2_Cl_2_, δ), 7.34–7.21 (m, 5H, Ar-H), 4.64–4.35 (m, 4H, ferrocenyl-H), 4.32 (s, 5H, ferrocenyl-H), 4.24–4.21 (m, 2H, CH_2_) ppm. ^13^C-NMR (CD_2_Cl_2_, δ), 137.0 (Ar-C), 129.1 (Ar-C), 128.8 (Ar-C), 127.8 (Ar-C), 72.9 (dd, *J*(P,C) = 11.6 Hz, ferrocenyl-C), 72.5 (d, *J*(P,C) = 13.6 Hz, ferrocenyl-C), 72.4 (d, *J*(P,C) = 19.7 Hz, ferrocenyl-C), 71.2 (d, *J*(P,C) = 15.4 Hz, ferrocenyl-C), 70.8 (s, ferrocenyl-C), 37.5 (SCH_2_) ppm. ^31^P-NMR (CD_2_Cl_2_, δ), 116.3 (d, *J*(P,F) = 1101 Hz) pm. ^19^F-NMR (CD_2_Cl_2_, δ), −39.1 (d, *J*(F,P) = 1103 Hz) ppm. MS (CI^+^, *m*/*z*), 391 [C_17_H_16_FFePS_2_H]^+^. Accurate mass measurement (CI^+^MS): 388.9880 [C_17_H_16_F^54^FePS_2_H]^+^, calculated mass for [C_17_H_16_F^54^FePS_2_H]^+^: 388.9884.

#### 3.2.10. Synthesis of Potassium3-fluoronaphtho[1,8-*cd*][1,2,6]oxadiphosphinine-1(3*H*)-thiolate 1,3-disulfide (**11**)

A mixture of 1,3-epithionaphtho[1,8-*cd*][1,2,6]oxadiphosphinine 1,3-disulfide (0.600 g, 2.0 mmol) and freshly dried and finely ground KF (0.232 g, 4.0 mmol) in dry acetonitrile (50 mL) was stirred at ambient temperature. After mixing for 10 min, the mixture was heated at 80 °C for 2 h. Upon cooling to room temperature the reaction mixture was filtered and the filtrate was evaporated *in vacuo* to give the title compound **11** as pale yellow solid (0.700 g, 98%). M.p. 170–171 °C. Selected IR (KBr, cm^−1^): 1494 (m), 1219 (m), 1159 (m), 1029 (m), 926 (vs), 847 (s), 821 (s), 762 (s), 682 (vs), 650 (vs), 584 (s), 561 (s), 438 (m). ^1^H-NMR (CD_3_OD, δ), 8.53 (dd, *J*(H,H) = 8.2 Hz, *J*(P,H) = 1.4 Hz, 1H, Ar-H), 8.48–8.44 (m, 1H, Ar-H), 8.21 (d, *J*(H,H) = 8.2 Hz, 1H, Ar-H), 8.04–8.01 (m, 1H, Ar-H), 7.70–7.61 (m, 2H, Ar-H) ppm. ^13^C-NMR (CD_3_OD, δ), 140.0 (d, *J*(P,C) = 95.5 Hz, Ar-C), 134.9 (s, Ar-C), 134.1 (d, *J*(P,C) = 14.5 Hz, Ar-C), 131.9 (d, *J*(P,C) = 15.6 Hz, Ar-C), 130.9 (s, Ar-C), 126.4 (s, Ar-C), 126.1 (s, Ar-C), 125.1 (s, Ar-C), 124.9 (s, Ar-C), 117.0 (s, Ar-C) ppm. ^31^P-NMR (CD_3_OD, δ), 107.1 (d, ^2^*J*(P,P) = 54.0 Hz), 78.1 (dd, *J*(P,F) = 1099 Hz, ^2^*J*(P,P) = 54.0 Hz) pm. ^19^F-NMR (CD_3_OD, δ), −42.6 (d, *J*(F,P) = 1104 Hz) ppm. MS (CI^−^, *m*/*z*), 319 [C_10_H_7_FOP_2_S_3_]^−^. Accurate mass measurement (CI^−^MS): 318.9030 [C_10_H_7_FOP_2_S_3_]^−^, calculated mass for [C_10_H_7_FOP_2_S_3_]^−^: 318.9040.

#### 3.2.11. Synthesis of Tetrabutylammonium 3-fluoronaphtho[1,8-*cd*][1,2,6]oxadiphosphinine-1(3*H*)-thiolate 1,3-disulfide (**12**)

To a suspension of 1,3-epithionaphtho[1,8-*cd*][1,2,6]oxadiphosphinine 1,3-disulfide (0.600 g, 2.0 mmol) was added dropwise tetrabutylammonium fluoride (4 mL of 1 M solution in THF, 4.0 mmol) at room temperature. The off-white suspension disappeared immediately and a brown clear solution formed. The mixture was stirred for one more hour for completion. After filtering through a Celit lawyer the filtrate was evaporated *in vacuo* to give the title compound **12** as brown sticky paste (1.902 g, 85%). Selected IR (KBr, cm^−1^): 1485 (s), 1381 (m), 1218 (m), 1162 (m), 947 (s), 904 (m), 852 (m), 772 (m), 695 (s), 642 (m), 587 (s), 552 (m). ^1^H-NMR (CD_2_Cl_2_, δ), 8.52–7.42 (m, 6H, Ar-H), 3.13 (t, *J*(H,H) = 8.0 Hz, 8H, CH_2_), 1.55–1.53 (m, 8H, CH_2_), 1.33 (q, *J*(H,H) = 8.0 Hz, 8H, CH_2_), 0.92 (t, *J*(H,H) = 8.0 Hz, 12H, CH_3_) ppm. ^13^C-NMR (CD_2_Cl_2_, δ), 142.0 (d, *J*(P,C) = 95.8 Hz, Ar-C), 134.8 (s, Ar-C), 134.1 (d, *J*(P,C) = 15.6 Hz, Ar-C), 131.7 (d, *J*(P,C) = 14.5 Hz, Ar-C), 130.4 (s, Ar-C), 128.9 (s, Ar-C), 126.7 (s, Ar-C), 126.4 (s, Ar-C), 125.2 (s, Ar-C), 124.9 (s, Ar-C), 58.7 (CH_2_), 23.9 (CH_2_), 19.7 (CH_2_), 13.5 (CH_3_) ppm. ^31^P-NMR (CD_2_Cl_2_, δ), 106.9 (d, ^2^*J*(P,P) = 54.0 Hz), 78.6 (dd, *J*(P,F) = 1099 Hz, ^2^*J*(P,P) = 54.0 Hz) pm. ^19^F-NMR (CD_2_Cl_2_, δ), −28.4 (dd, ^1^*J*(F,P) = 1104 Hz, ^3^*J*(F,P) = 3.2 Hz) ppm. MS (CI^+^, *m*/*z*), 242 [C_16_H_36_N]^+^. Accurate mass measurement (CI^+^MS): 242.2841 [C_16_H_36_N]^+^, calculated mass for [C_16_H_36_N]^+^: 242.2842; MS (CI^−^, *m*/*z*), 319 [C_10_H_7_FOP_2_S_3_]^−^. Accurate mass measurement (CI^−^MS): 318.9032 [C_10_H_7_FOP_2_S_3_]^−^, calculated mass for [C_10_H_7_FOP_2_S_3_]^−^: 318.9040.

#### 3.2.12. Synthesis of Tetraphenylphosphonium3-fluoronaphtho[1,8-*cd*][1,2,6]oxadiphosphinine-1(3*H*)-thiolate 1,3-disulfide (**13**)

Potassium3-fluoronaphtho[1,8-*cd*][1,2,6]oxadiphosphinine-1(*3H*)-thiolate 1,3-disulfide (0.358 g. 1.0 mmol) was dissolved in degassed water (10 mL). To this solution was added dropwise an equivalent amount of tetraphenylphosphonium chloride (0.374 g, 1.0 mmol) in water (10 mL). The precipitate almost immediately formed and the mixture was allowed to continue stirring for 1 h. The salt was harvested by filtration and was dried over 100 °C *in vacuo* to give the title compound **13** as off-white solid (0.446 g, 68%). M.p. 163–165 °C. Selected IR (KBr, cm^−1^): 1585 (m), 1481 (m), 1436 (s), 1185 (m), 1163 (m), 1107 (vs), 922 (s), 826 (s), 753 (s), 72 3(s), 689 (vs), 647 (s), 583 (s), 526 (vs). ^1^H-NMR (CD_2_Cl_2_, δ), 8.52–8.31 (m, 2H, Ar-H), 8.09 (d, *J*(H,H) = 8.3 Hz, 1H, Ar-H), 7.91–7.85 (m, 4H, Ar-H), 7.75–7.71 (m, 7H, Ar-H), 7.64–7.56 (m, 12H, Ar-H) ppm. ^13^C-NMR (CD_2_Cl_2_, δ), 141.9 (d, *J(*P,C) = 96.0 Hz, Ar-C), 135.7 (d, *J*(P,C) = 3.2 Hz, Ar-C), 134.6 (s, Ar-C), 134.5 (s, Ar-C), 134.4 (Ar-C), 134.0 (Ar-C), 133.9 (Ar-C), 131.7 (Ar-C), 131.6 (Ar-C), 130.6 (d, *J*(P,C) = 12.5 Hz, Ar-C), 130.1 (Ar-C), 126.5 (d, *J*(P,C) = 10.8 Hz, Ar-C), 124.9 (d, *J*(P,C) = 12.5 Hz, Ar-C), 117.9 (d, Ar-C), 117.1 (d, Ar-C) ppm. ^31^P-NMR (CD_2_Cl_2_, δ), 106.9 (d, ^2^*J*(P,P) = 54.0 Hz), 78.2 (dd, *J*(P,F) = 1099 Hz, ^2^*J*(P,P) = 54.0 Hz), 23.8 (s) pm. ^19^F-NMR (CD_2_Cl_2_, δ), −28.7 (dd, ^1^*J*(F,P) = 1100 Hz, ^3^*J*(F,P) = 3.2 Hz) ppm. MS (CI^+^, *m*/*z*), 339 [C_24_H_20_P]^+^. Accurate mass measurement (CI^+^MS): 339.1296 [C_24_H_20_P]^+^, calculated mass for [C_24_H_20_P]^+^: 339.1297. MS (CI^−^, *m*/*z*), 319 [C_10_H_7_FOP_2_S_3_]^−^. Accurate mass measurement (CI^−^MS): 318.9031 [C_10_H_7_FOP_2_S_3_]^−^, calculated mass for [C_10_H_7_FOP_2_S_3_]^−^: 318.9040.

#### 3.2.13. Synthesis of 1,3,4-Triphenyl-1*H*-1,2,4-triazol-4-ium3-fluoronaphtho[1,8-*cd*][1,2,6]oxadiphosphinine-1(3*H*)-thiolate 1,3-disulfide (**14**)

Potassium3-fluoronaphtho[1,8-*cd*][1,2,6]oxadiphosphinine-1(3*H*)-thiolate 1,3-disulfide (0.096 g, 0.27 mmol) was dissolved in degassed water (5 mL). To this solution was added dropwise an equivalent of 1,3,4-triphenyl-1*H*-1,2,4-triazol-4-ium tetrafluoroborate (0.103 g, 0.27 mmol) in water/acetone (1:1, 10 mL). The mixture solution was stirred at room temperature for 2 h. Then, the organic solvent was evaporated under reduced pressure and the pale yellow precipitate was almost immediately out. After filtration and washing with water (2 × 5 mL) and ethyl ether (5 mL), the precipitate was dried at 100 °C in vacuum to give the title compound **14** as brown solid (0.156 g, 94%). M.p. 154–155 °C. Selected IR (KBr, cm^−1^): 1586 (m), 1559 (s), 1485 (s), 1460 (m), 1447 (m), 1340 (m), 1239 (m), 1061 (s), 936 (s), 841 (m), 762 (s), 687 (vs), 644 (s), 584 (s). ^1^H-NMR (CD_2_Cl_2_, δ), 11.20 (s, 1H, triazol-H), 8.37–8.07 (m, 3H, Ar-H), 7.66–7.39 (m, 18H, Ar-H) ppm. ^13^C-NMR (CD_2_Cl_2_, δ), 154.0 (triazol-C), 142.0 (triazol-C), 135.0 (Ar-C), 134.8 (Ar-C), 134.5 (Ar-C), 134.2 (Ar-C), 132.7 (Ar-C), 131.7 (Ar-C), 131.2 (Ar-C), 130.9 (Ar-C), 130.5 (Ar-C), 130.2 (Ar-C), 129.8 (Ar-C), 129.7 (Ar-C), 129.5 (Ar-C), 129.3 (Ar-C), 128.7 (Ar-C), 128.5 (Ar-C), 128.2 (Ar-C), 126.6 (Ar-C), 126.3 (Ar-C), 125.3 (Ar-C), 125.0 (Ar-C), 124.4 (Ar-C), 122.0 (Ar-C), 121.3 (Ar-C) ppm. ^31^P-NMR (CD_2_Cl_2_, δ), 106.5 (d, ^2^J(P,P) = 54.0 Hz), 78.6 (dd, J(P,F) = 1106 Hz, ^2^J(P,P) = 54.0 Hz) pm. ^19^F-NMR (CD_2_Cl_2_, δ), −28.5 (d, J(F,P) = 1106 Hz) ppm. MS (CI^+^, *m*/*z*), 298 [C_20_H_16_N_3_]^+^. Accurate mass measurement (CI^+^MS): 298.1335 [C_20_H_16_N_3_]^+^, calculated mass for [C_20_H_16_N_3_]^+^: 298.1339; MS (CI^−^, *m*/*z*), 319 [C_10_H_7_FOP_2_S_3_]^−^. Accurate mass measurement (CI^−^MS): 318.9031 [C_10_H_7_FOP_2_S_3_]^−^, calculated mass for [C_10_H_7_FOP_2_S_3_]^−^: 318.9040.

#### 3.2.14. Synthesis of 1,3-Dimesityl-4,5-dihydro-1*H*-imidazol-3-ium3-fluoronaphtho[1,8-*cd*][1,2,6]oxadiphosphinine-1(3*H*)-thiolate 1,3-disulfide (**15**)

Potassium3-fluoronaphtho[1,8-*cd*][1,2,6]oxadiphosphinine-1(3*H*)-thiolate 1,3-disulfide (0.108 g, 0.30 mmol) was dissolved in degassed water (10 mL). To this solution was added dropwise an equivalent amount of 1,3-dimesityl-4,5-dihydro-1*H*-imidazol-3-ium chloride (0.102 g, 0.30 mmol) in water (10 mL). The precipitation was almost immediately formed and the mixture was allowed to continue stirring for 2 h. The precipitate was harvested by filtration and dissolved in dichloromethane (30 mL). The solution was dried over MgSO_4_ and dried *in vacuo* to give the title compound **15** as white foam (0.175 g, 93%). Selected IR (KBr, cm^−1^): 1628 (vs), 1481 (m), 1378 (m), 1261 (s), 1217 (m), 1162 (m), 942 (s), 904 (m), 849 (m), 82 5(m), 769 (m), 691 (s), 643 (s), 585 (s). ^1^H-NMR (CD_2_Cl_2_, δ), 8.61 (s, 2H, Ar-H), 8.32–7.34 (m, 6H, Ar-H), 7.00 (s, 2H, Ar-H), 4.49 (s, 1H, imidazol-H), 3.66 (t, 2H, CH_2_), 2.36 (s, 12H, CH_3_), 2.32 (s, 6H, CH_3_), 1.80 (t, 3H, CH_2_) ppm. ^13^C-NMR (CD_2_Cl_2_, δ), 159.7 (imidazol-C ), 140.9 (Ar-C), 135.1 (Ar-C), 134.6 (Ar-C), 34.2 (Ar-C), 134.0 (Ar-C), 131.7 (Ar-C), 131.5 (Ar-C), 130.3 (Ar-C), 130.1 (Ar-C), 129.6 (Ar-C), 126.5 (Ar-C), 126.3 (Ar-C), 125.1 (Ar-C), 124.9 (Ar-C), 53.1 (imidazol-C), 51.8 (imidazol-C), 20.9 (CH_3_), 17.9 (CH_3_) ppm. ^31^P-NMR (CD_2_Cl_2_, δ), 106.4 (d, ^2^J(P,P) = 54.0 Hz), 78.2 (dd, J(P,F) = 1101 Hz, ^2^J(P,P) = 54.0 Hz) pm. ^19^F-NMR (CD_2_Cl_2_, δ), −28.7 (dd, ^1^J(F,P) = 1100 Hz, ^3^J(P,F) = 3.3 Hz) ppm. MS (CI^+^, *m*/*z*), 307 [C_21_H_27_N_2_]^+^. Accurate mass measurement (CI^+^MS): 307.2170 [C_21_H_27_N_2_]^+^, calculated mass for [C_21_H_27_N_2_]^+^: 307.2169; MS (CI^−^, *m*/*z*), 319 [C_10_H_7_FOP_2_S_3_]^−^. Accurate mass measurement (CI^−^MS): 318.9031 [C_10_H_7_FOP_2_S_3_]^−^, calculated mass for [C_10_H_7_FOP_2_S_3_]^−^: 318.9040.

#### 3.2.15. Synthesis of 1,5-Epoxynaphtho[1,8-*cd*][1,7,2,6]dithiadiphosphocine 1,5-disulfide (**18**)

A mixture of potassium3-fluoronaphtho[1,8-*cd*][1,2,6]oxadiphosphinine-1(*3H*)-thiolate 1,3-disulfide (0.101 g, 0.30 mmol) or tetrabutylammonium ferrocenylphosphonofluoridodithioate (0.162 g, 0.30 mmol) and dry dichloromethane (10 mL) was stirred at room temperature for 48 h. After removing the unreacted solid by filtration the remaining filtrate was concentrated to 2 mL and was purified by column (eluted by dichloromethane) to give the title compound **18** as yellow foam (0.048 g, 46%) for both starting materials **11** and **12**. M.p. 78–80 °C. Selected IR (KBr, cm^−1^): 1584 (m), 1483 (m), 1436 (s), 1317 (m), 1186 (m), 1159 (m), 1107 (s), 996 (m), 890 (m), 825 (m), 768 (m), 723 (s), 687 (s), 590 (s), 525 (vs), 453 (m). ^1^H-NMR (CD_2_Cl_2_, δ), 7.91–7.84 (m, 2H, Ar-H), 7.76–7.69 (m, 2H, Ar-H), 7.63–7.55 (m, 2H, Ar-H) ppm. ^13^C-NMR (CD_2_Cl_2_, δ), 141.7 (d, *J*(P,C) = 3.0 Hz, Ar-C), 138.6 (d, *J*(P,C) = 10.8 Hz, Ar-C), 134.3 (d, *J*(P,C) = 13.0 Hz, Ar-C), 130.5 (d, *J*(P,C) = 117.9 Hz, Ar-C), 129.9 (Ar-C), 124.6 (Ar-C) ppm. ^31^P-NMR (CD_2_Cl_2_, δ), 106.9 (d, ^2^*J*(P,P) = 54.0 Hz) pm. MS (EI^+^, *m*/*z*), 348 [C_10_H_6_O_2_P_2_S_4_]^+^. Accurate mass measurement (EI^+^MS): 347.8719 [C_10_H_6_O_2_P_2_S_4_]^+^, calculated mass for [C_10_H_6_O_2_P_2_S_4_]^+^: 347.8727.

#### 3.2.16. Synthesis of Potassium (4-phenoxyphenyl)phosphonofluoridodithioate (**19**)

A mixture of 2,4-bis(4-phenoxyphenyl)-1,3,2,4-dithiadiphosphetane 2,4-disulfide (Belleau’s Reagent) (1.056 g, 2.0 mmol) and freshly dried and finely ground KF (0.232 g, 4.0 mmol) in dry acetonitrile (50 mL) was stirred at ambient temperature. After mixing for 10 min, the mixture was heated at 80 °C for 2 h. Upon cooling to room temperature the reaction mixture was filtered and the filtrate was evaporated *in vacuo* to give the title compound **19** as pale yellow paste (1.250 g, 97%). Selected IR (KBr, cm^−1^): 1596 (s), 1571 (m), 1501 (s), 1406 (m), 1296 (s), 1253 (s), 1181 (m), 1112 (s), 1022 (s), 973 (m), 829 (m), 688 (s), 556 (s), 385 (m). ^1^H-NMR (CD_3_OD, δ), 8.07–7.96 (m, 1H, Ar-H), 7.94–7.86 (m, 2H, Ar-H), 7.80–7.70 (m, 2H, Ar-H), 6.84–6.70 (m, 2H, Ar-H) ppm. ^13^C-NMR (CD_3_OD, δ), 163.6 (Ar-C), 163.1 (Ar-C), 132.2 (d, *J*(P,C) = 112.1 Hz, Ar-C), 131.3 (d, *J*(P,C) = 13.5 Hz, Ar-C), 118.2 (Ar-C), 116.9 (s, Ar-C), 112.6 (Ar-C), 112.3 (s, Ar-C) ppm. ^31^P-NMR (CD_3_OD, δ), 130.2 (d, *J*(P,F) = 1028 Hz) pm. ^19^F-NMR (CD_3_OD, δ), −26.3 (d, *J*(F,P) = 1030 Hz) ppm. MS (CI^−^, *m*/*z*), 283 [C_12_H_9_FOPS_2_]^−^. Accurate mass measurement (CI^−^MS): 283.2954 [C_12_H_9_FOPS_2_]^−^, calculated mass for [C_12_H_9_FOPS_2_]^−^: 283.2951.

#### 3.2.17. Synthesis of Tetrabutylammonium (4-phenoxyphenyl)phosphonofluoridodithioate (20)

To a suspension of 2,4-bis(4-phenoxyphenyl)-1,3,2,4-dithiadiphosphetane 2,4-disulfide (Belleau’s Reagent) (0.528 g, 1.0 mmol) in THF (30 mL) was added dropwise tetrabutylammonium fluoride (2 mL of 1 M solution in THF, 2.0 mmol) at room temperature. The off-white suspension disappeared immediately and a colorless solution formed. The mixture was stirred for one more hour for completion. After filtering through a Celit lawyer, the filtrate was evaporated in vacuum to give the title compound **20** as slightly greenish yellow oil (0.520 g, 99%). Selected IR (KBr, cm^−1^): 1595 (s), 1499 (s), 1426 (m), 1292 (m), 1252 (s), 1177 (m), 1108 (s), 1027 (m), 831 (m), 800 (m), 738 (m), 691 (s), 554 (m). ^1^H-NMR (CD_2_Cl_2_, δ), 8.08–7.91 (m, 5H, Ar-H), 6.87–6.81 (m, 4H, Ar-H), 3.23–3.04 (m, 8H, NCH_2_), 1.63–1.51 (m, 8H, CH_2_), 1.37 (m, 8H, CH_2_), 0.98 (t, *J*(H,H) = 7.3 Hz, 12H, CH_3_) ppm. ^13^C-NMR (CD_2_Cl_2_, δ), 162.2, 160.9, 136.5 (d, *J*(P,C) = 112.0 Hz, Ar-C), 131.6 (d, *J*(P,C) = 13.6 Hz, Ar-C), 131.4 (s, Ar-C), 113.0 (s, Ar-C), 112.8 (s, Ar-C), 112.5 (s, Ar-C), 58.8 (CH_2_), 24.0 (CH_2_), 19.7 (CH_2_), 13.4 (CH_3_) ppm. ^31^P-NMR (CD_2_Cl_2_, δ), 126.3 (d, *J*(P,F) = 1016 Hz) pm. ^19^F-NMR (CD_2_Cl_2_, δ), −20.9 (d, *J*(F,P) = 1017 Hz) ppm. MS (CI^+^, *m*/*z*), 242 [C_16_H_36_N]^+^. Accurate mass measurement (CI^+^MS): 242.2841 [C_16_H_36_N]^+^, calculated mass for [C_16_H_36_N]^+^: 242.2842; MS (CI^−^, *m*/*z*), 283 [C_12_H_9_FOPS_2_]^−^. Accurate mass measurement (CI^−^MS): 283.2953 [C_12_H_9_FOPS_2_]^−^, calculated mass for [C_12_H_9_FOPS_2_]^−^: 283.2951.

#### 3.2.18. Synthesis of Tetraphenylphosphonium (4-phenoxyphenyl)phosphonofluoridodithioate (**21**)

Potassium (4-phenoxyphenyl)phosphonofluoridodithioate (0.322 g. 1.0 mmol) was dissolved in degassed water/acetone (1:1, 30 mL). To this solution was added dropwise an equivalent amount of tetraphenylphosphonium chloride (0.374 g, 1.0 mmol) in water (10 mL). The mixture was allowed to continue stirring at room temperature for 3 h. After removing the organic solvent, the mixture was extracted with dichloromethane (2 × 30 mL). The organic lawyer was washed with water and dried over MgSO_4_ to give the title compound **21** as white form (0.534 g, 86%) after removing the solvent and drying *in vacuo*. Selected IR (KBr, cm^−1^): 1594 (m), 1497 (m), 1438 (m), 1250 (m), 1179 (m), 1108 (s), 1024 (m), 830 (m), 800 (m), 723 (s), 688 (s), 554 (m), 526 (s). ^1^H-NMR (CD_2_Cl_2_, δ), 8.04–7.87 (m, 6H, Ar-H), 7.77–7.73 (m, 10H, Ar-H), 7.65–7.57 (m, 10H, Ar-H), 6.79–6.76 (m, 3H, Ar-H) ppm. ^13^C-NMR (CD_2_Cl_2_, δ), 160.7, 135.8 (d, *J*(P,C) = 3.1 Hz, Ar-C), 134.6 (Ar-C), 134.5 (Ar-C), 132.7 (Ar-C), 132.6 (Ar-C), 131.3 (d, *J*(P,C) = 14.5 Hz, Ar-C), 130.7 (d, *J*(P,C) = 13.5 Hz, Ar-C), 119.3 (Ar-C), 116.9 (Ar-C), 112.6 (Ar-C), 112.3 (Ar-C) ppm. ^31^P-NMR (CD_2_Cl_2_, δ), 126.4 (d, *J*(P,F) = 1019 Hz), 23.8 (s) pm. ^19^F-NMR (CD_2_Cl_2_, δ), −35.3 (d, *J*(F,P) = 1018 Hz) ppm. MS (CI^+^, *m*/*z*), 339 [C_24_H_20_P]^+^. Accurate mass measurement (CI^+^MS): 339.1297 [C_24_H_20_P]^+^, calculated mass for [C_24_H_20_P]^+^: 339.1297; MS (CI^−^, *m*/*z*), 283 [C_12_H_9_FOPS_2_]^−^. Accurate mass measurement (CI^−^MS): 283.2952 [C_12_H_9_FOPS_2_]^−^, calculated mass for [C_12_H_9_FOPS_2_]^−^: 283.2951.

## 4. Conclusions

In summary, the reaction of the ferrocene analogue of Lawesson reagent, 2,4-diferrocenyl-1,3,2,4-diathiadiphosphetane 2,4-disulfide {[FcP(μ-S)S]_2_, **FcLR**} with dry KF or tetrabutylammonium fluoride, led to the corresponding potassium and tetrabutylammonium salts of phenyldithiofluorophosphinic acids in excellent yields. Potassium phenyldithiofluorophosphinic acid is readily converted into the corresponding organic adduct by treating it with an equimolar amount of tetraphenylphosphonium chloride, and treating potassium phenyldiselenofluorophosphinic acid with mono- and di-halogenated alkanes gave rise of a series of the corresponding esters of phenylphosphonofluoridodithioates in high yields. Furthermore, using 1,3-epithionaphtho[*1*,*8*-*cd*][1,2,6]oxadiphosphinine 1,3-disulfide or Belleau’s reagent in its place resulted in the formation of a series of novel salts, adducts, and ester derivatives.

## Supplementary Materials

Supplementary materials can be accessed at: http://www.mdpi.com/1420-3049/20/07/12175/s1.
