# Supplementary material for: Novel Fluorinated Phosphorus–Sulfur Heteroatom Compounds: Synthesis and Characterization of Ferrocenyl- and Aryl-Phosphonofluorodithioic Salts, Adducts, and Esters
Source: Molecules. 2015 Jul 3;20(7):12175–97. doi: 10.3390/molecules200712175 (PMC6332468; doi:10.3390/molecules200712175)
Supplement: Supplementary file 1 [file molecules-20-12175-s001.pdf]

# Supplementary Materials

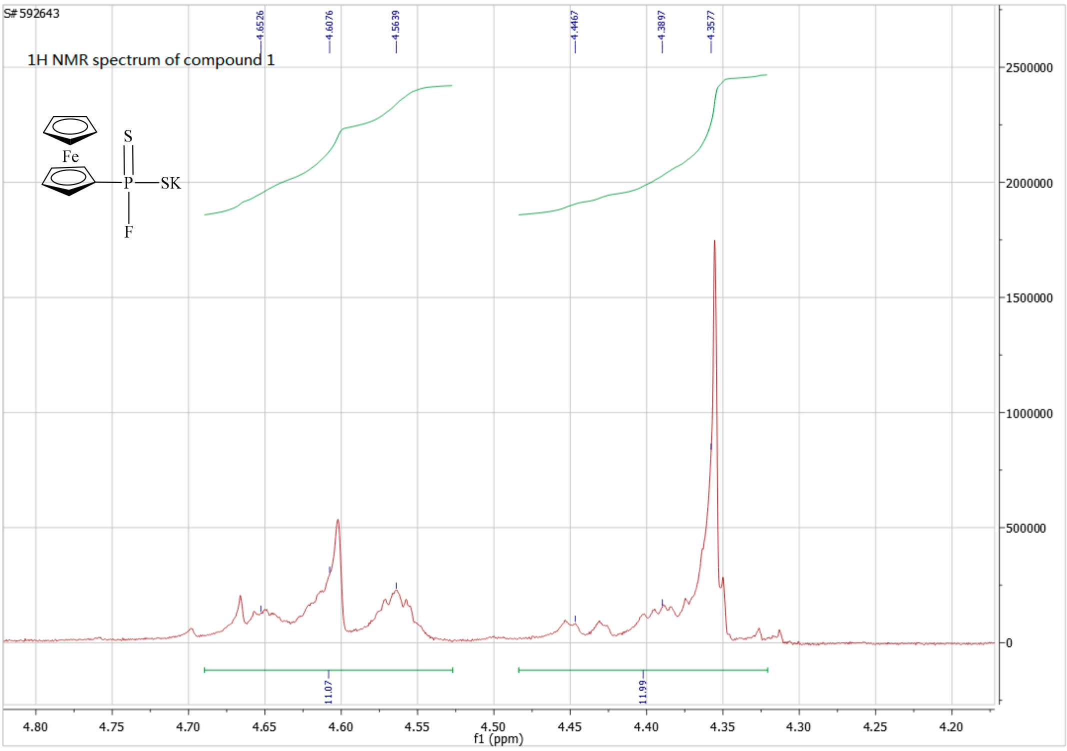


**Figure S1.** $^{1}$H-NMR spectra of compound **1**.

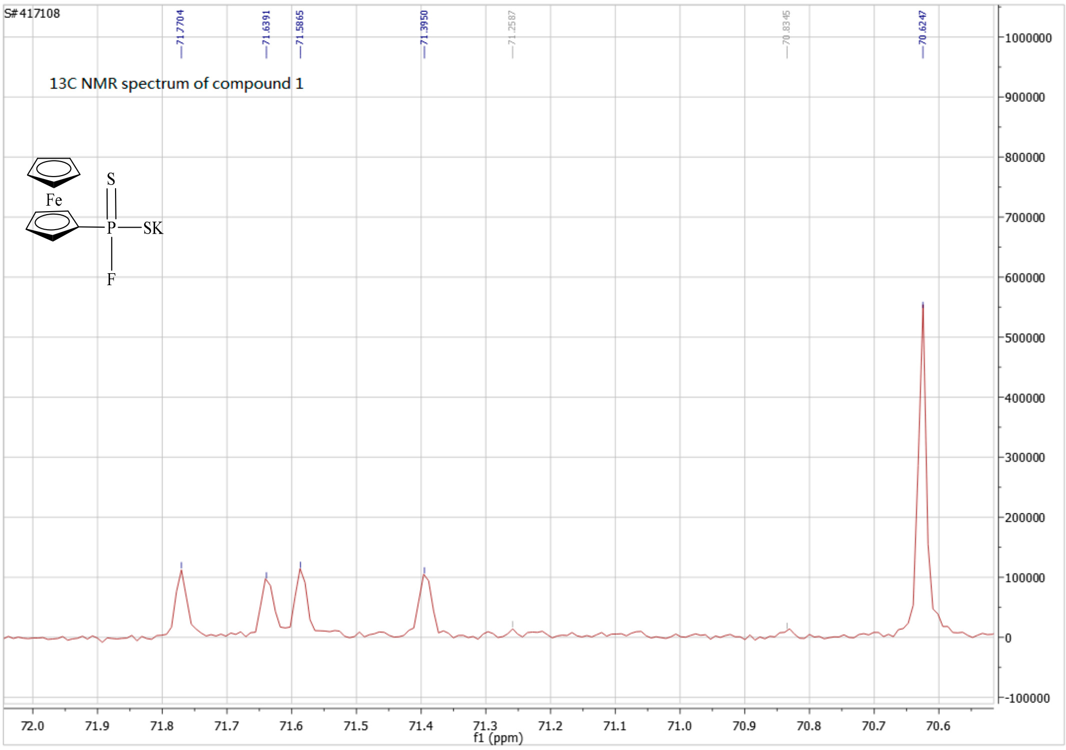


**Figure S2.** $^{13}$C-NMR spectra of compound **1**.

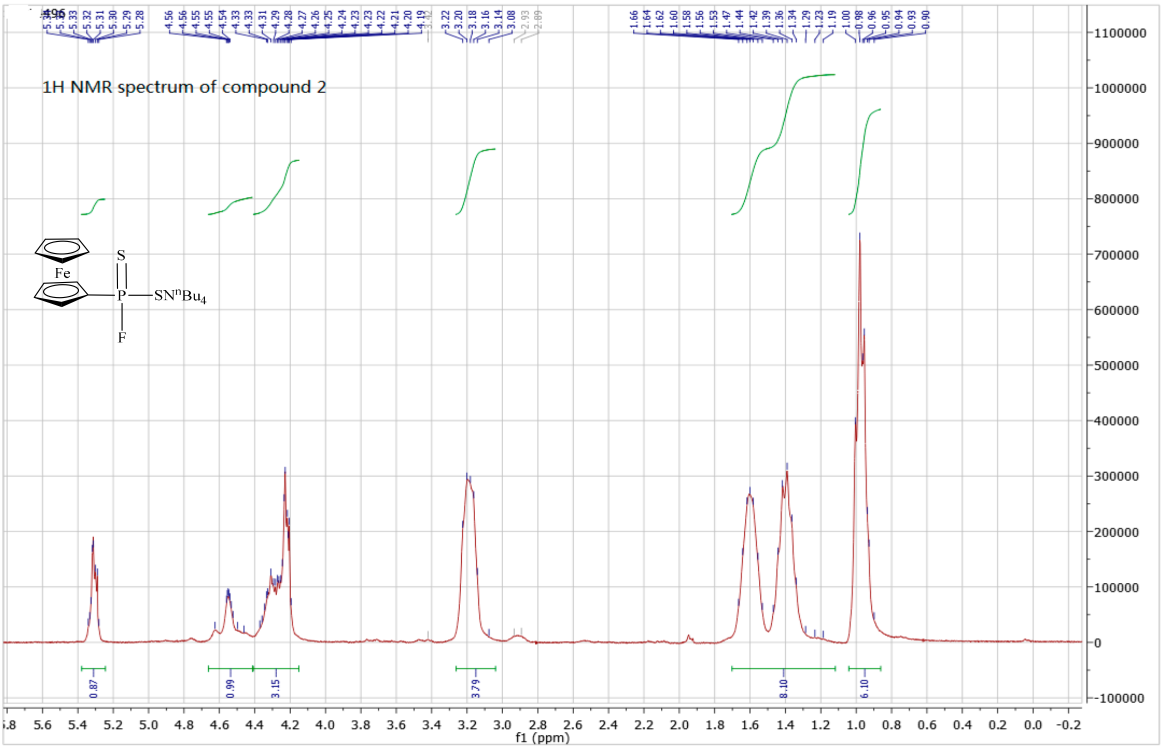


**Figure S3.** $^{1}$H-NMR spectra of compound **2**.

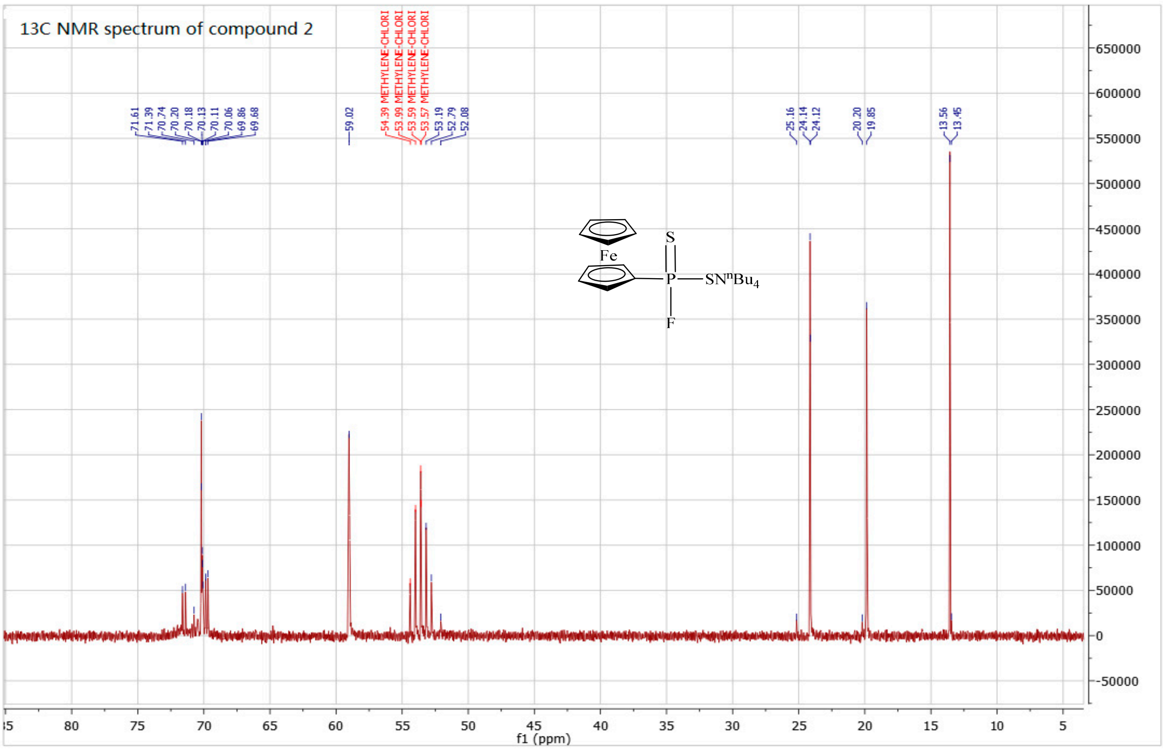


**Figure S4.** $^{13}$C-NMR spectra of compound **2**.

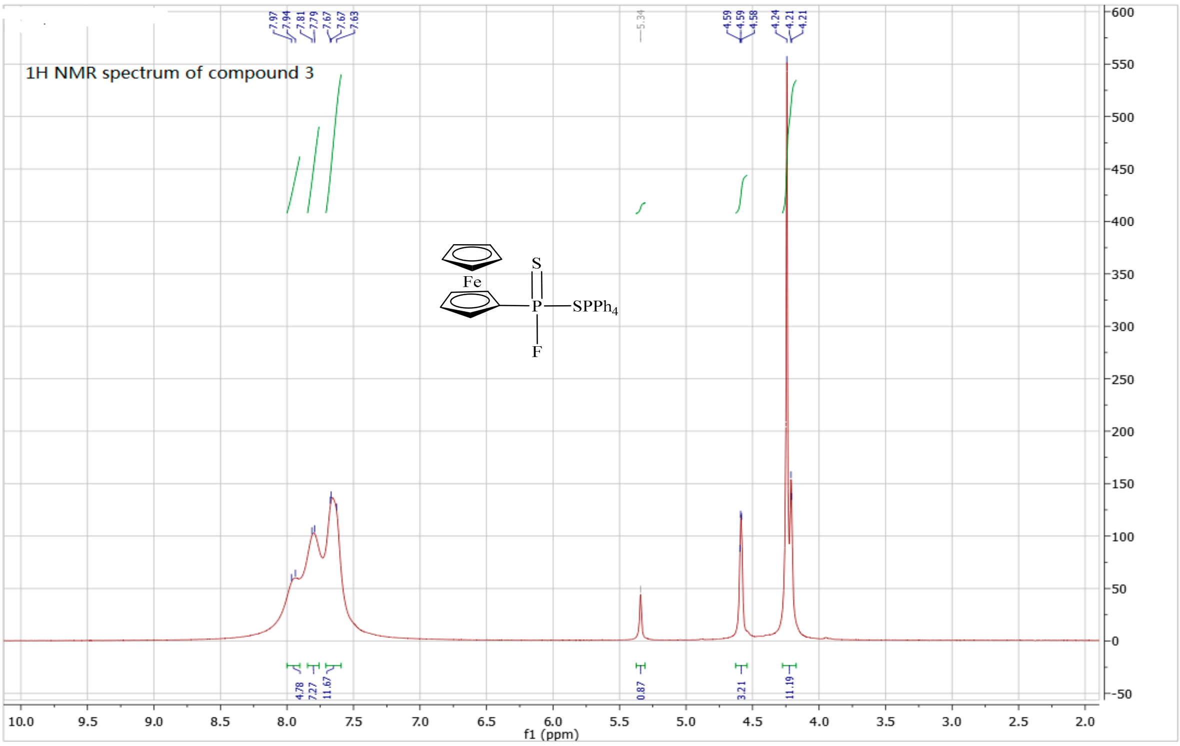


**Figure S5.** $^{1}$H-NMR spectra of compound **3**.

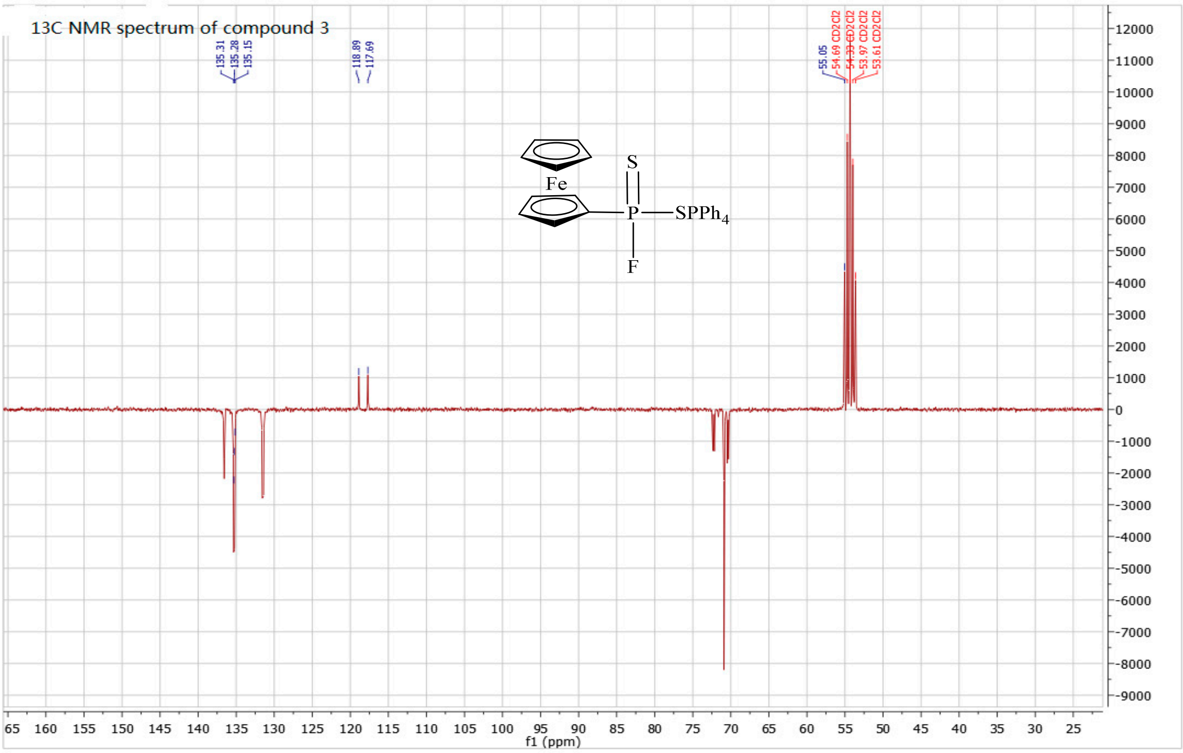


**Figure S6.** $^{13}$C-NMR spectra of compound **3**.

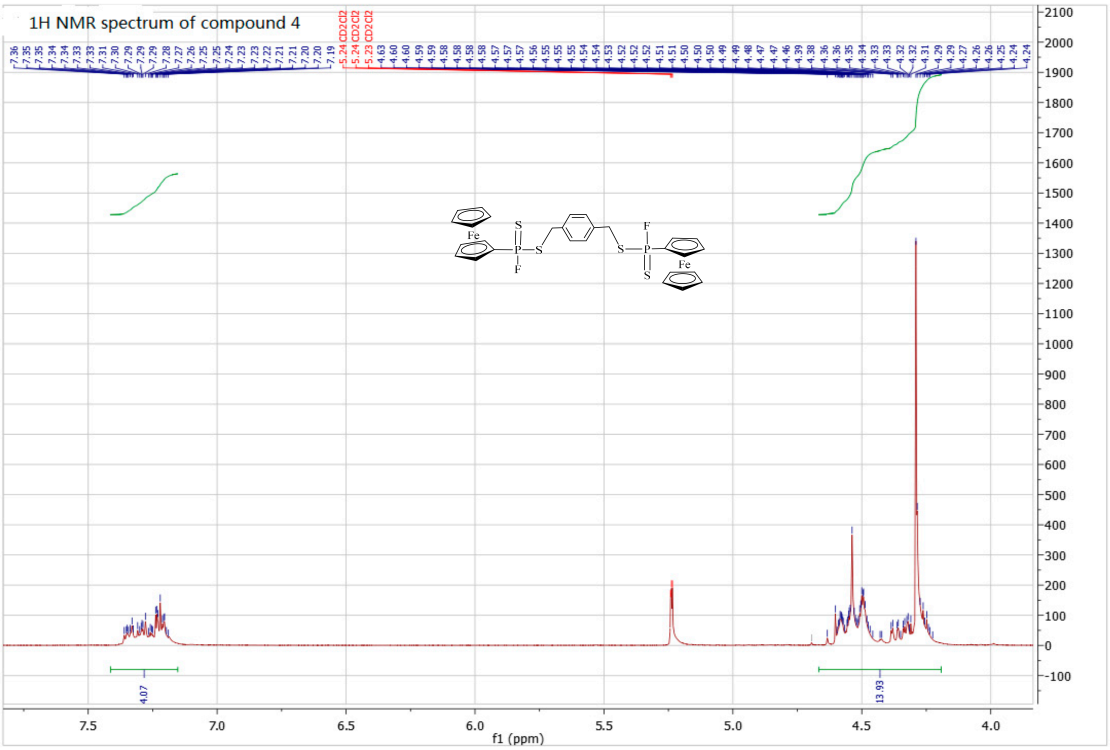


**Figure S7.** $^{1}$H-NMR spectra of compound **4**.

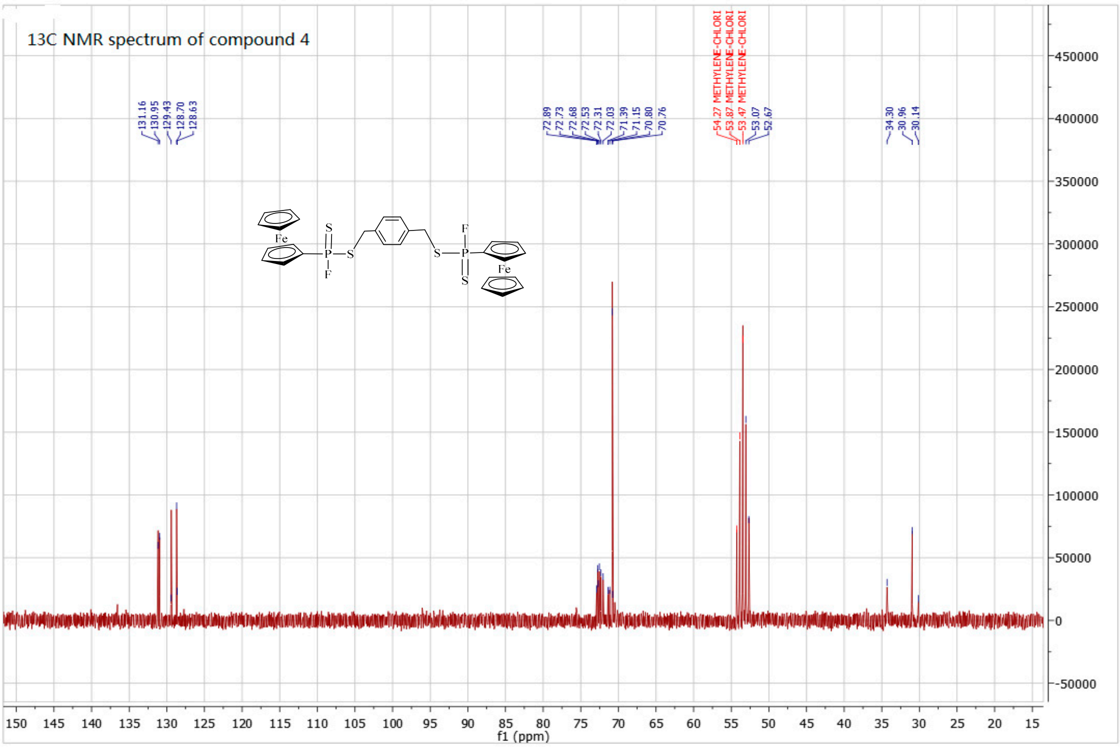


**Figure S8.** $^{13}$C-NMR spectra of compound **4**.

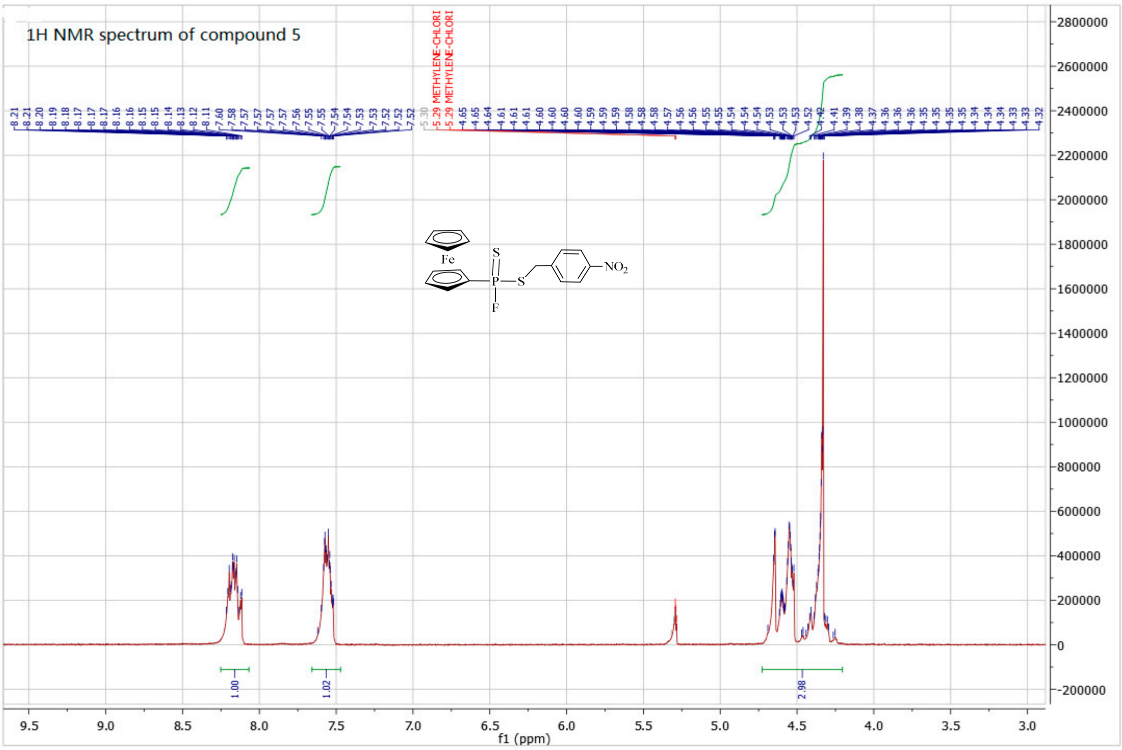


**Figure S9.** $^{1}$H-NMR spectra of compound **5**.

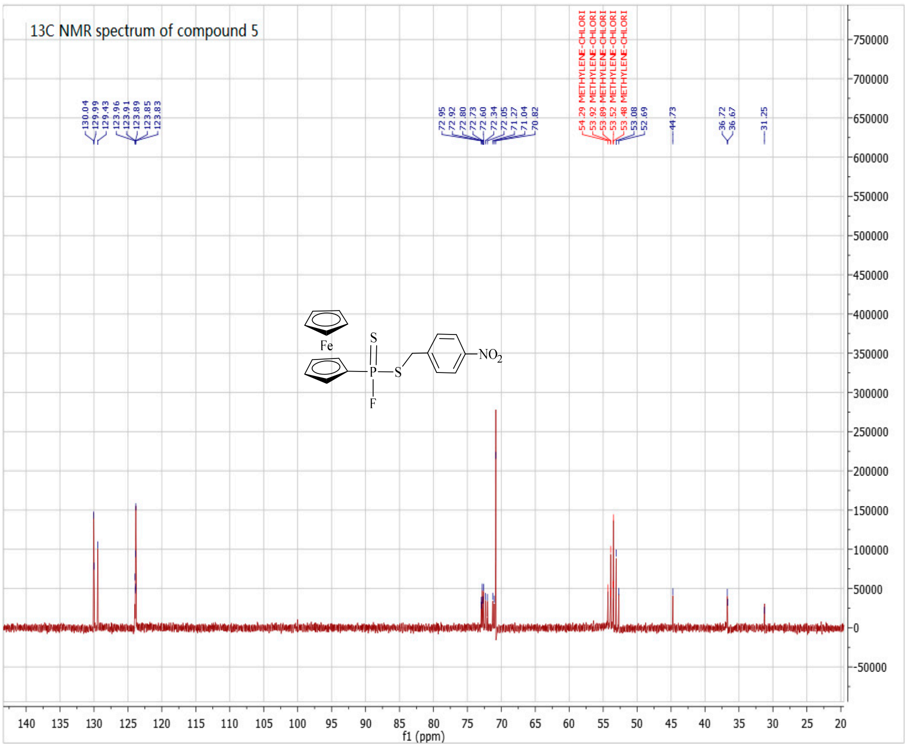


**Figure S10.** $^{13}$C-NMR spectra of compound **5**.

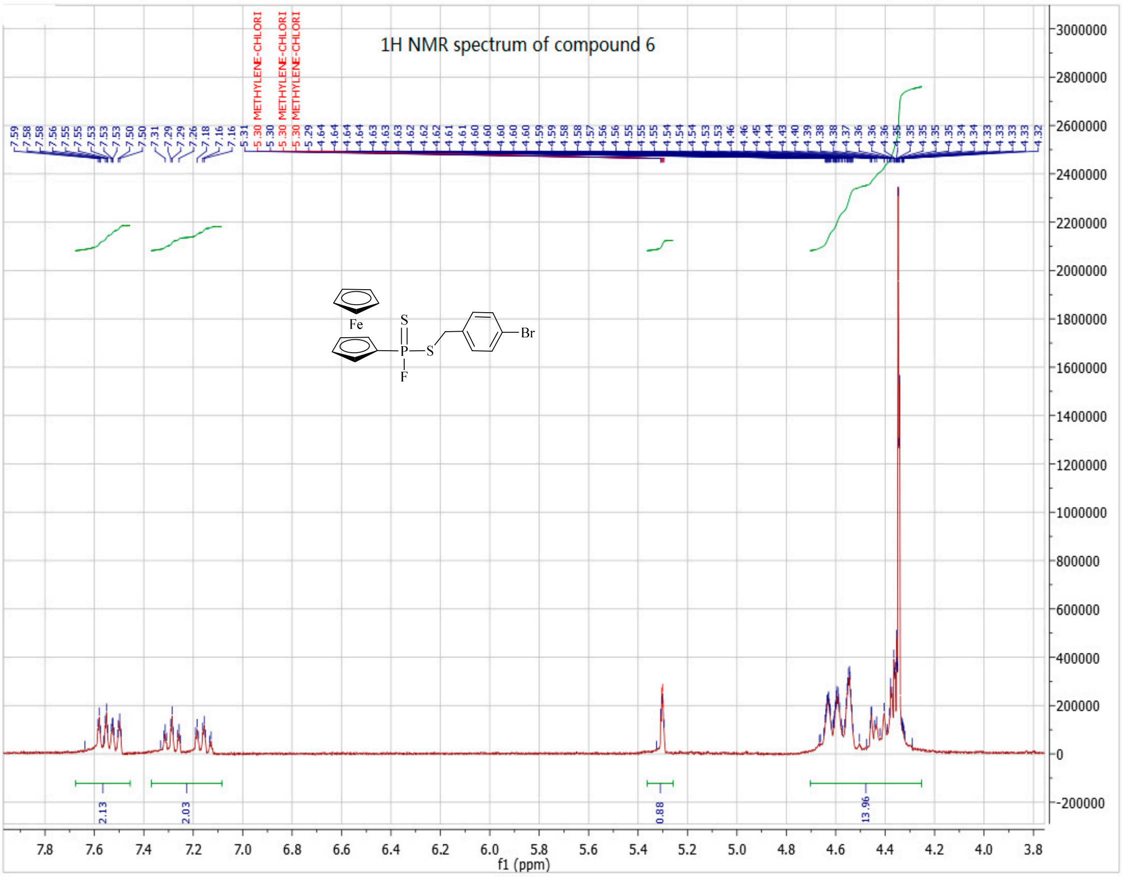


**Figure S11.** $^{1}$H-NMR spectra of compound **6**.

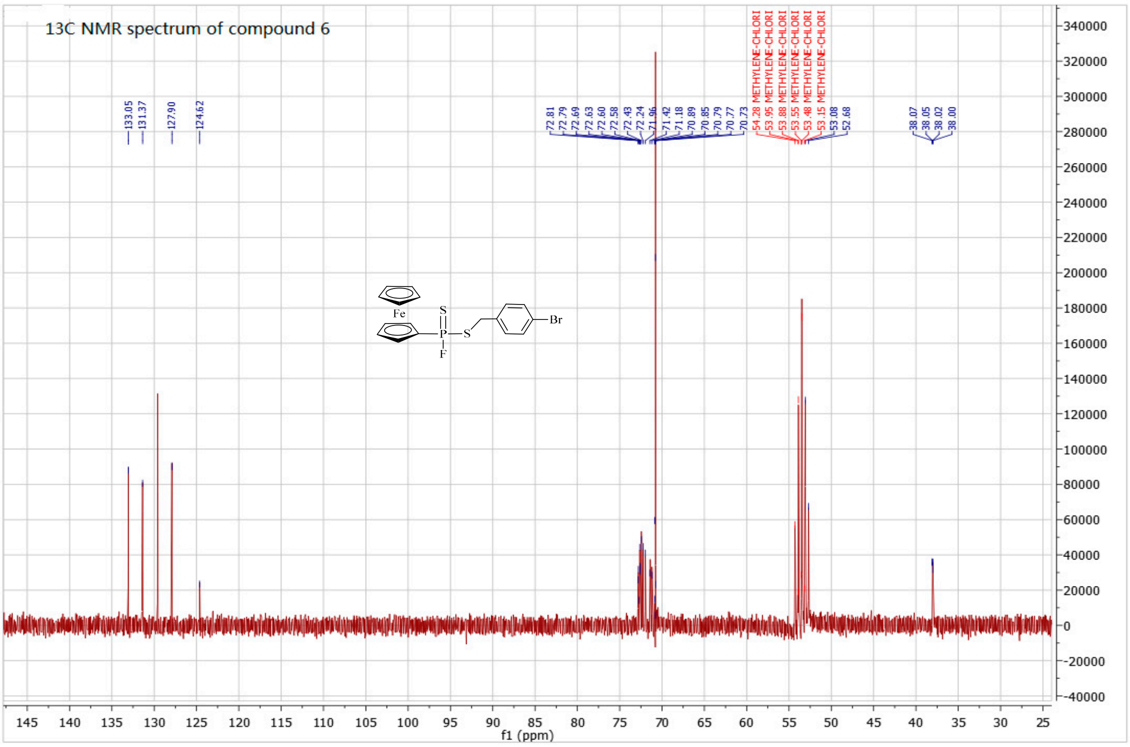


**Figure S12.** $^{13}$C-NMR spectra of compound **6**.

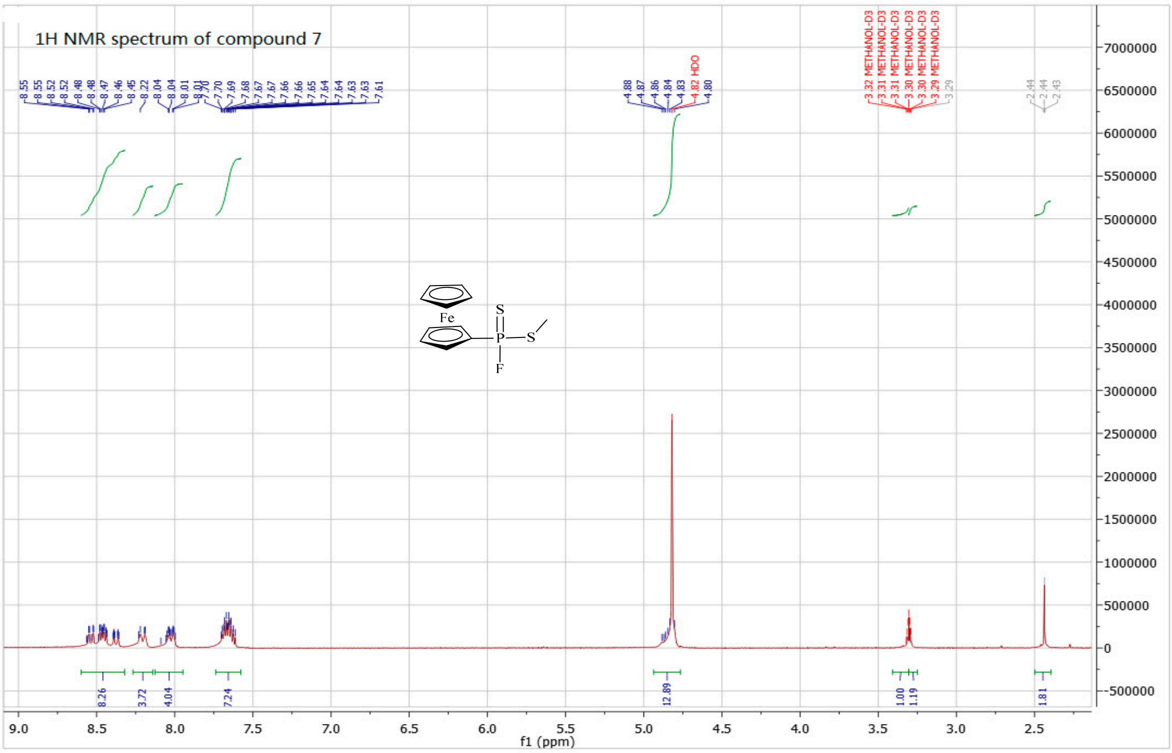


**Figure S13.** $^{1}$H-NMR spectra of compound **7.**

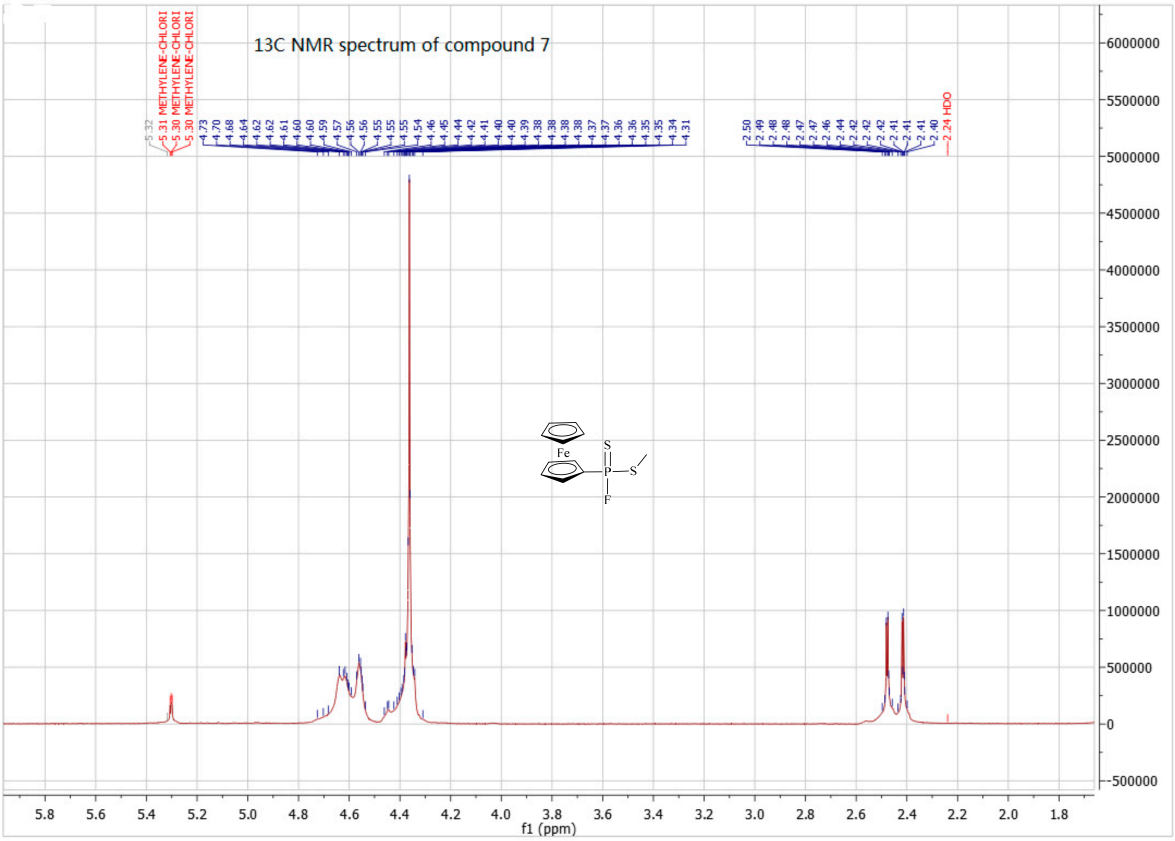


**Figure S14.** $^{13}$C-NMR spectra of compound **7**.

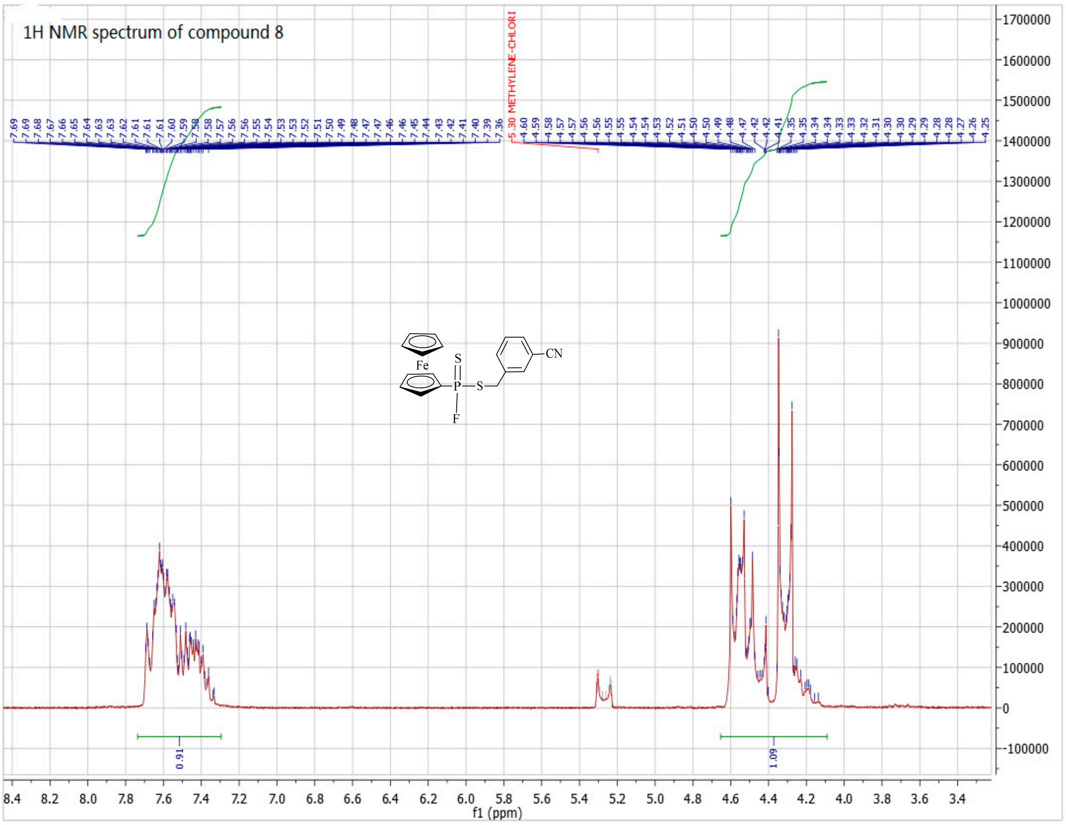


**Figure S15.** $^{1}$H-NMR spectra of compound **8**.

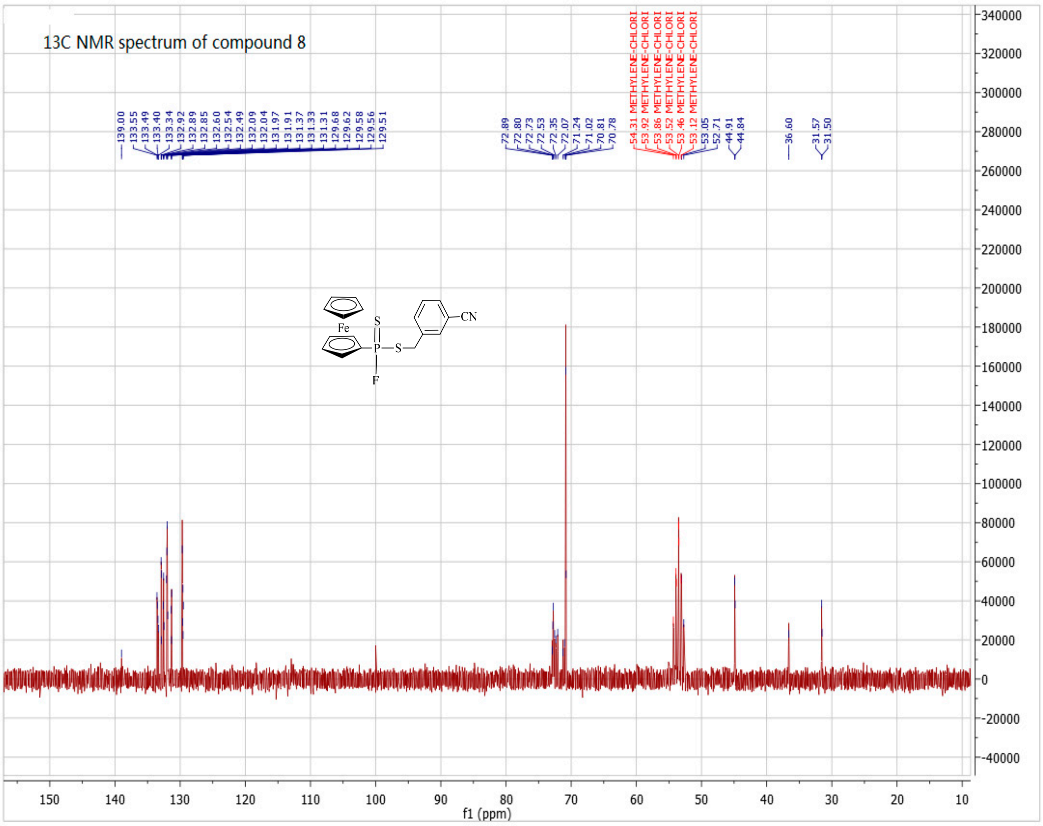


**Figure S16.** $^{13}$C-NMR spectra of compound **8**.

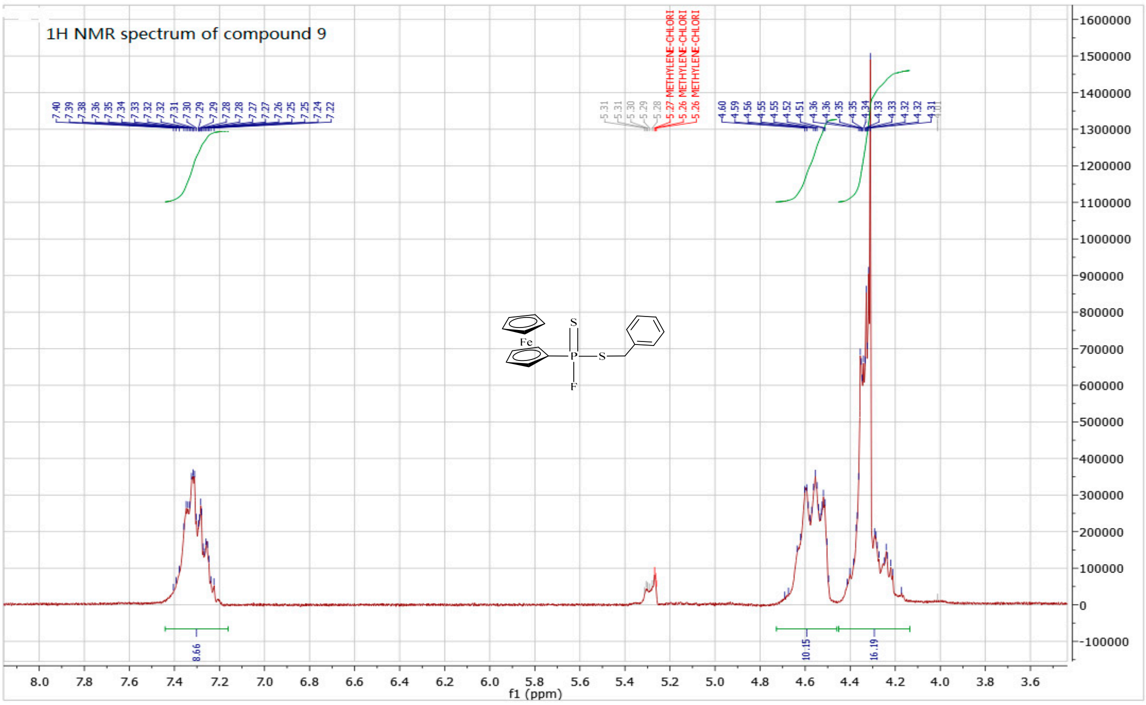


**Figure S17.** $^{1}$H-NMR spectra of compound **9**.

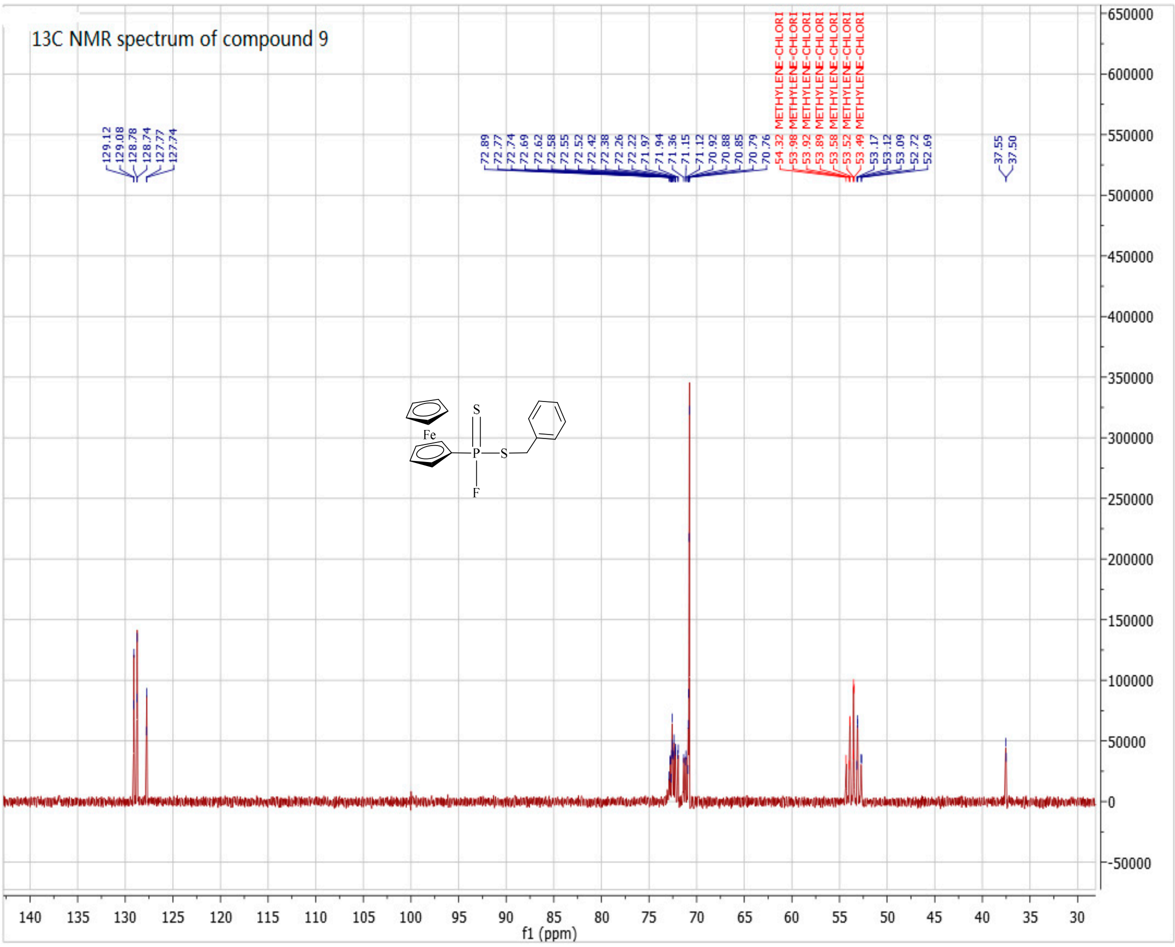


**Figure S18.** $^{13}$C-NMR spectra of compound **9**.

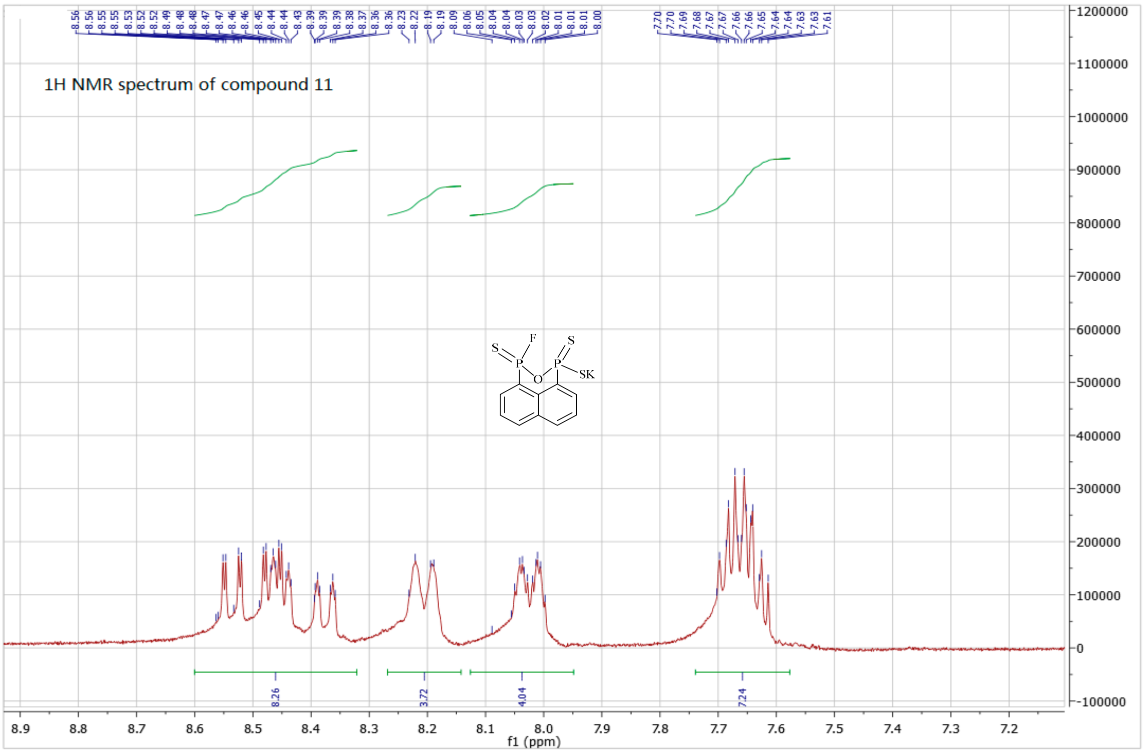


**Figure S19.** $^{1}$H-NMR spectra of compound **11**.

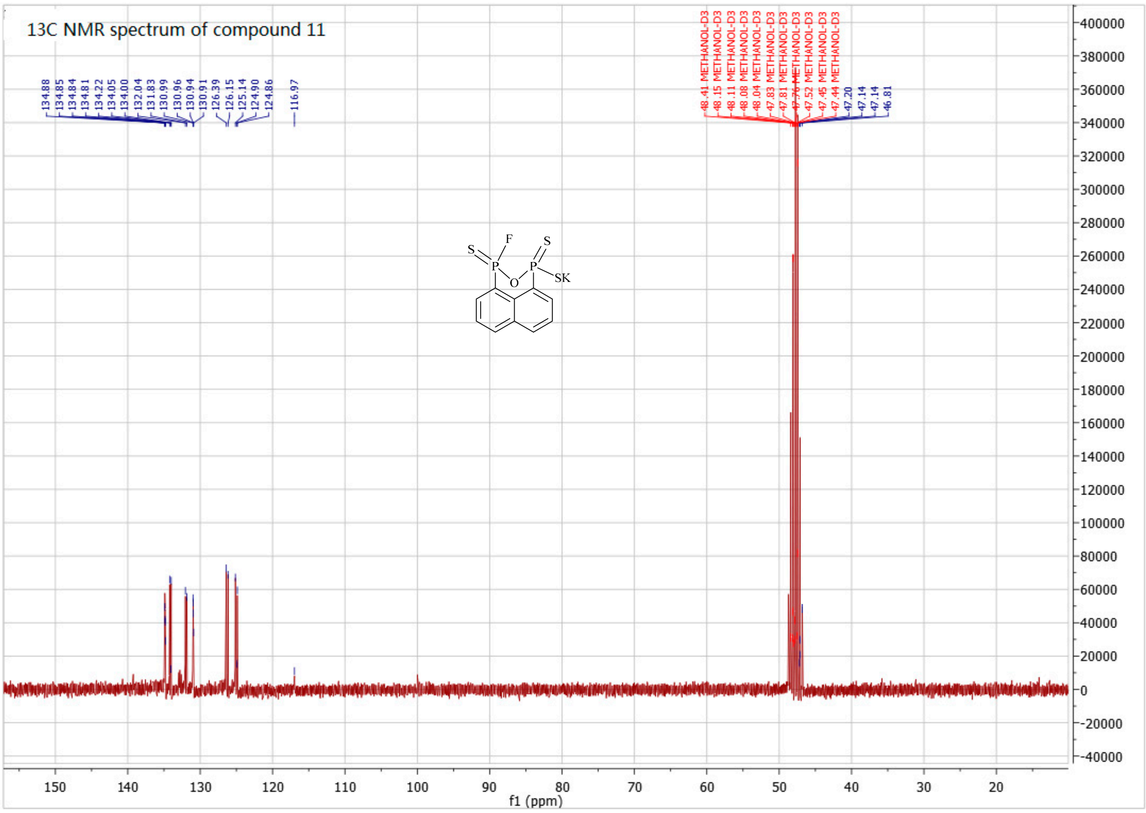


**Figure S20.** $^{13}$C-NMR spectra of compound **11**.

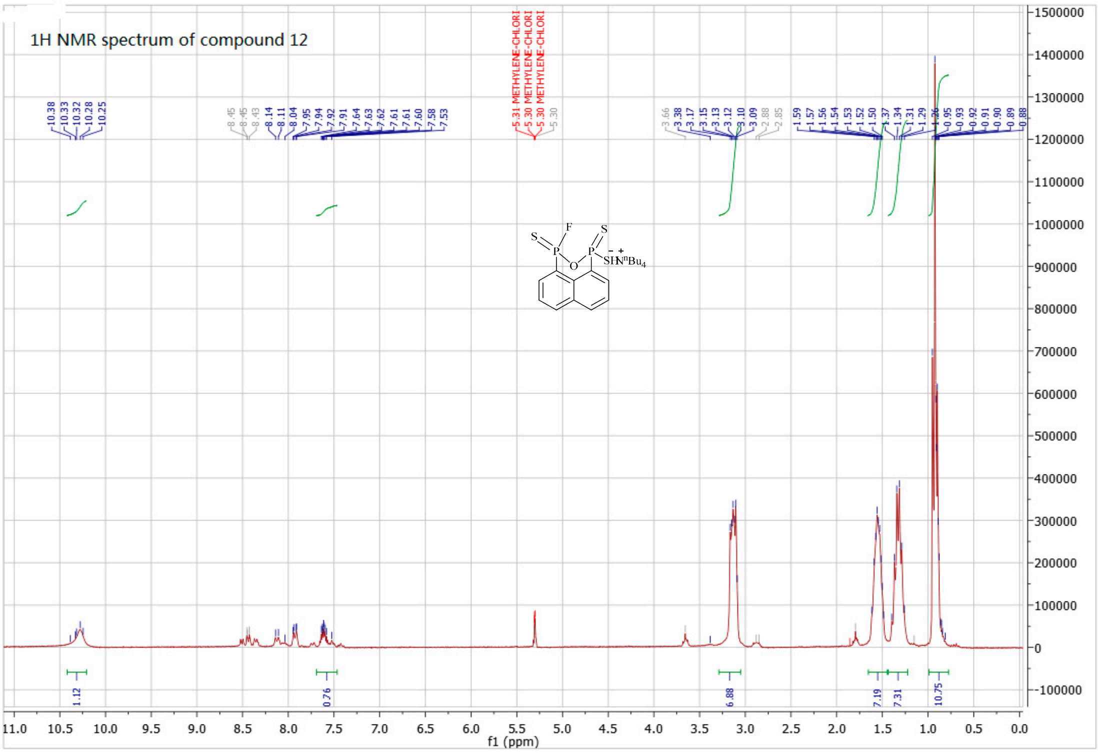


**Figure S21.** $^{1}$H-NMR spectra of compound **12**.

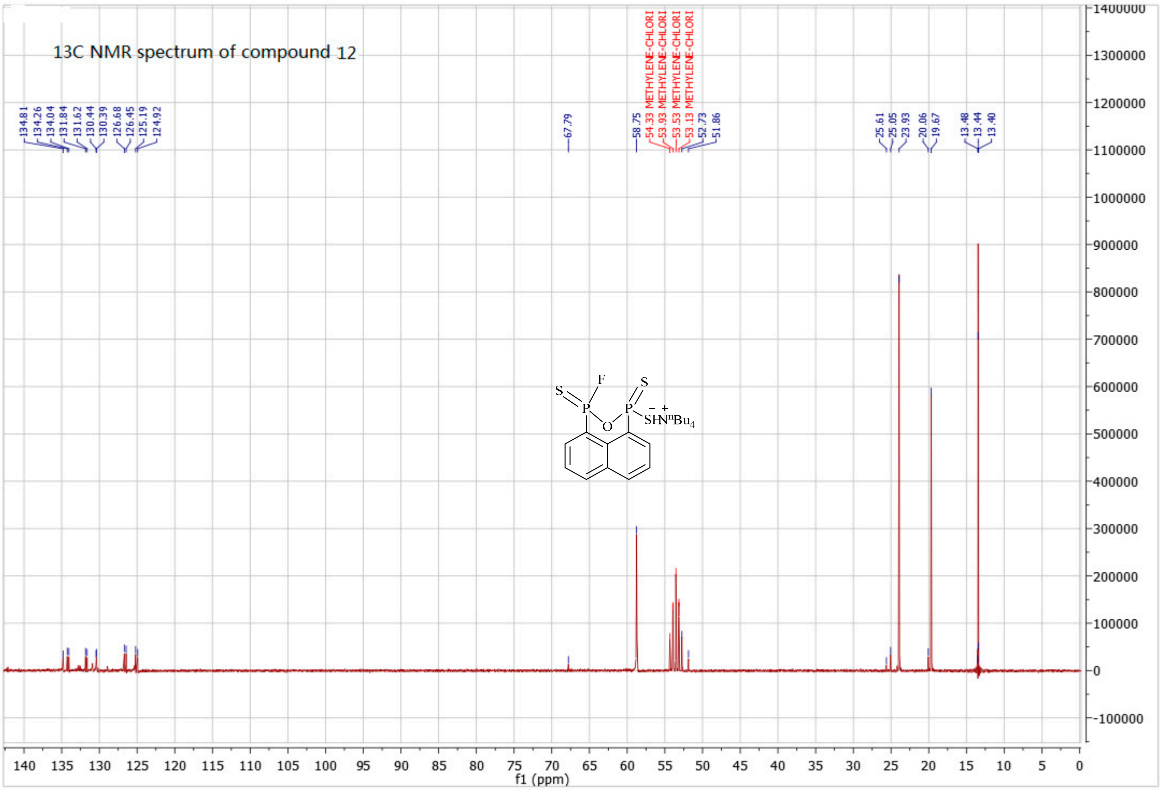


**Figure S22.** $^{13}$C-NMR spectra of compound **12**.

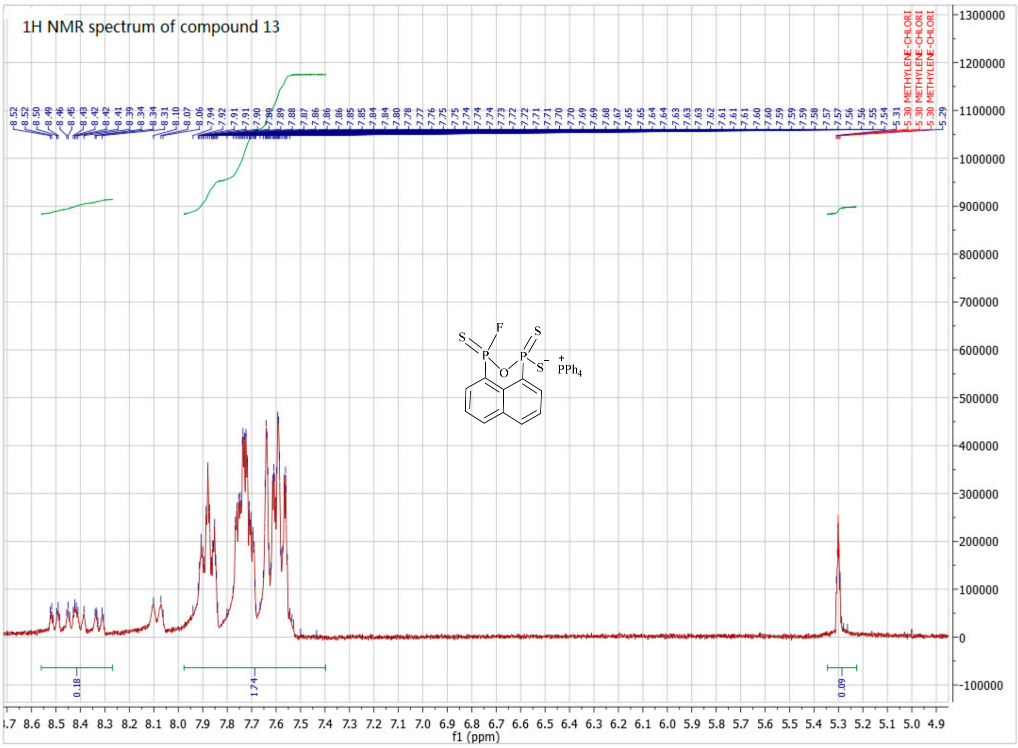


**Figure S23.** $^{1}$H-NMR spectra of compound **13**.

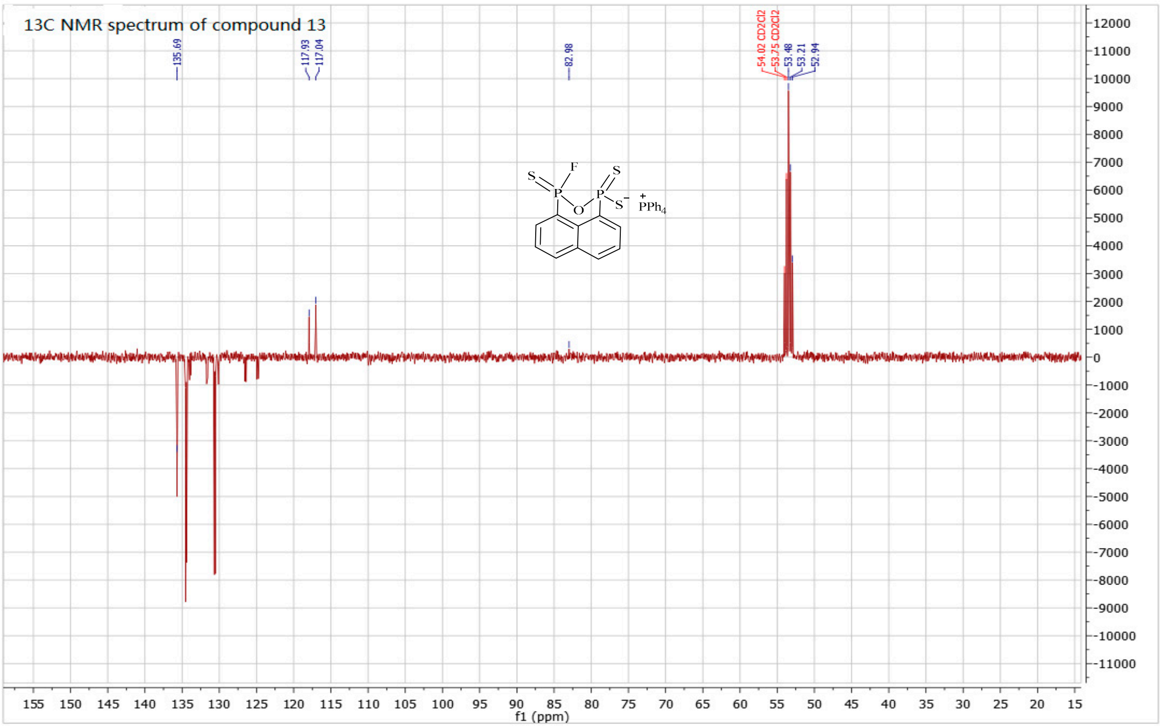


**Figure S24.** $^{13}$C-NMR spectra of compound **13**.

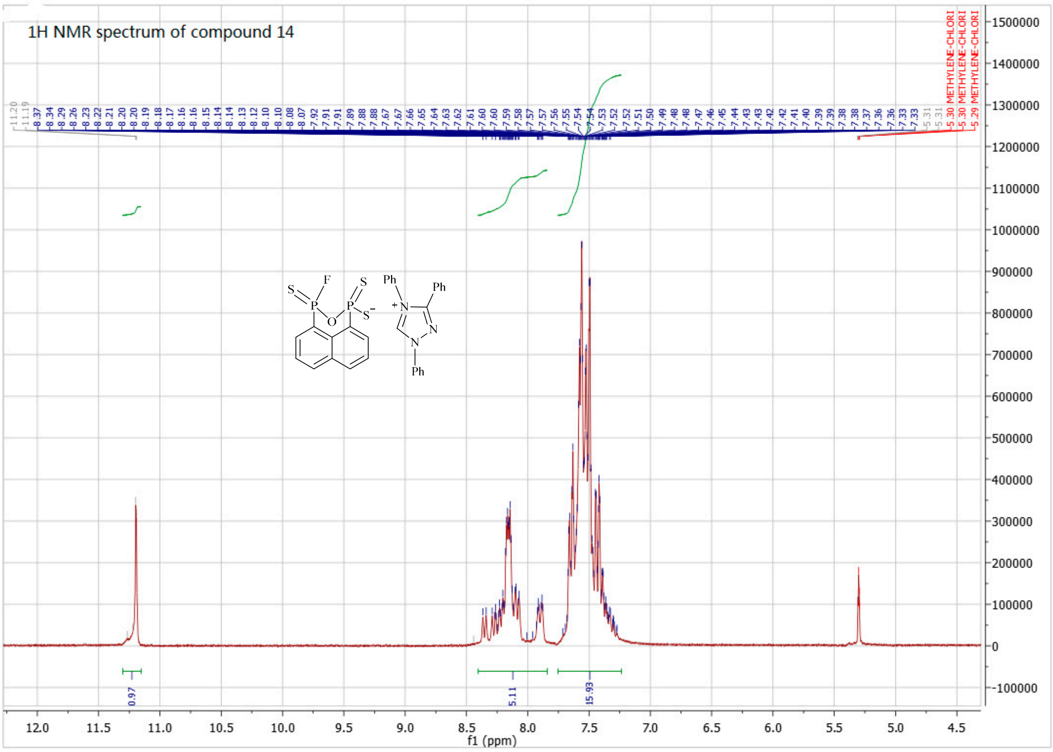


**Figure S25.** $^{1}$H-NMR spectra of compound **14**.

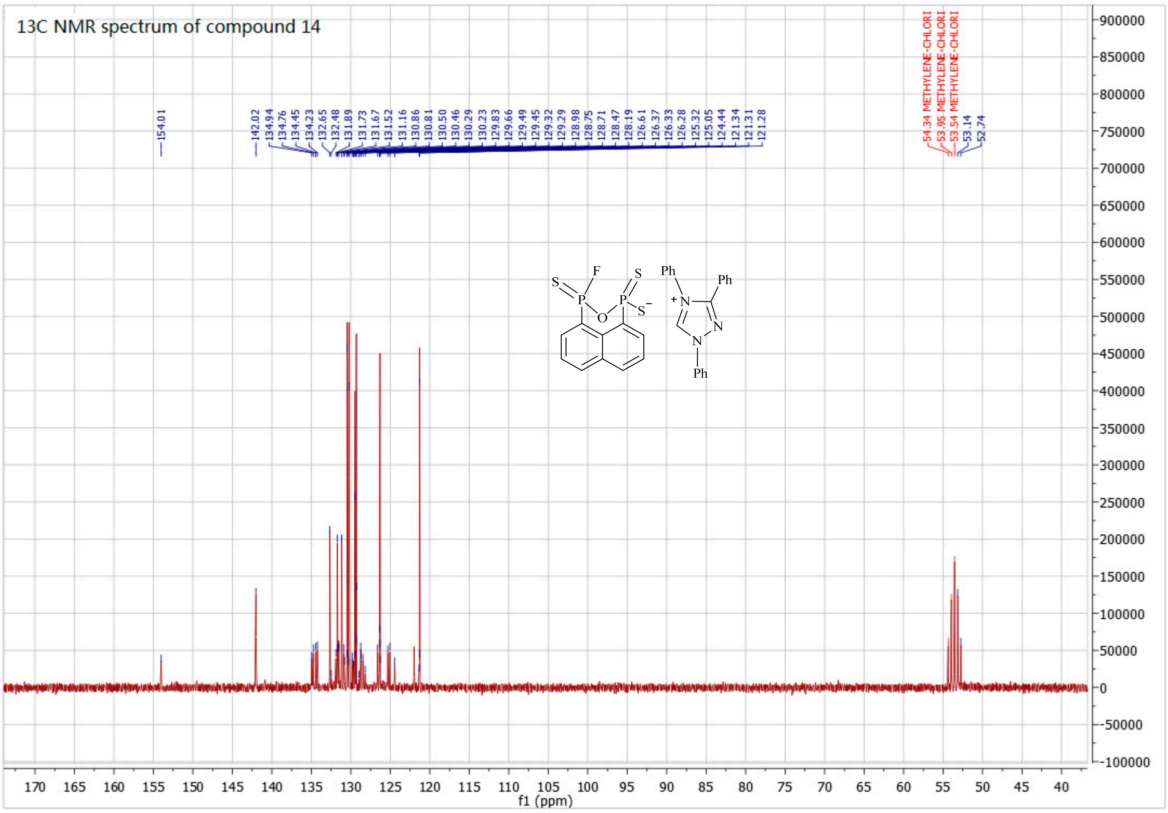


**Figure S26.** $^{13}$C-NMR spectra of compound **14**.

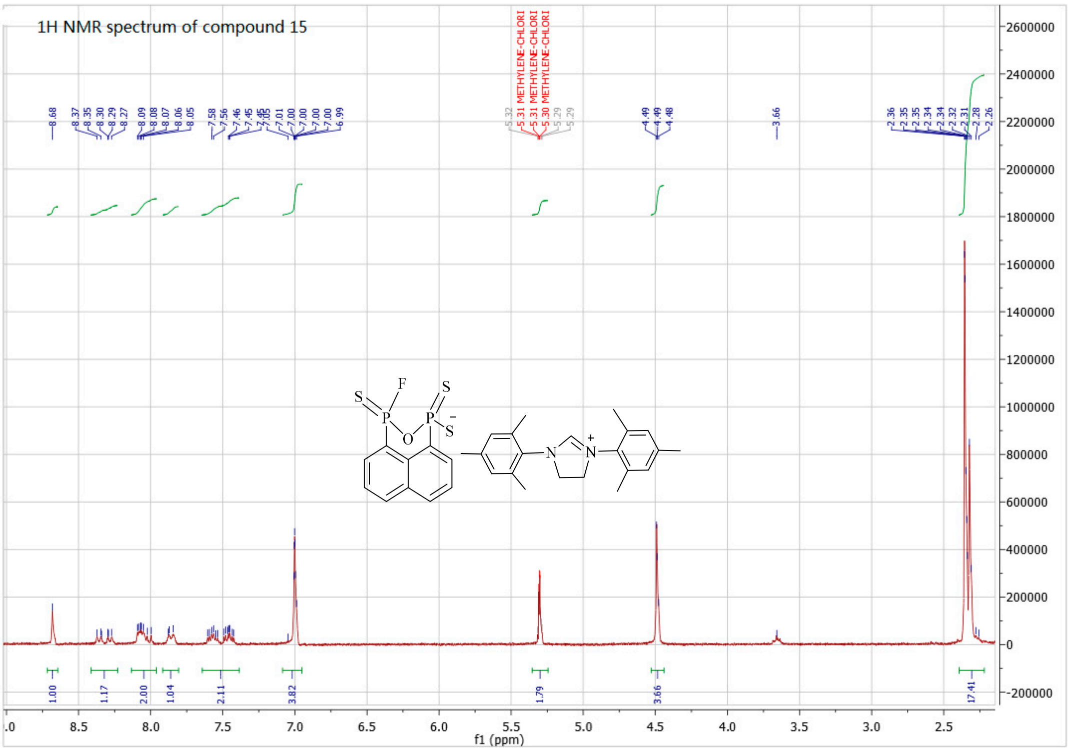


**Figure S27.** $^{1}$H-NMR spectra of compound **15**.

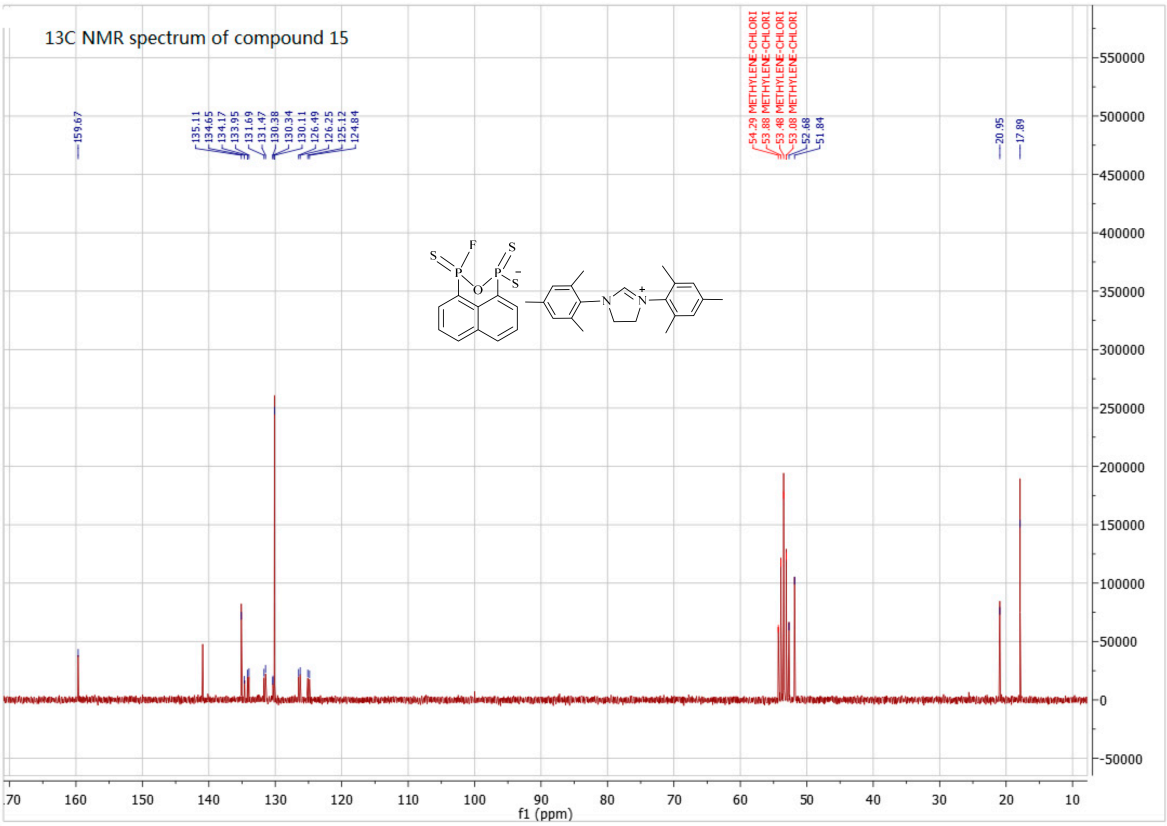


**Figure S28.** $^{13}$C-NMR spectra of compound **15**.

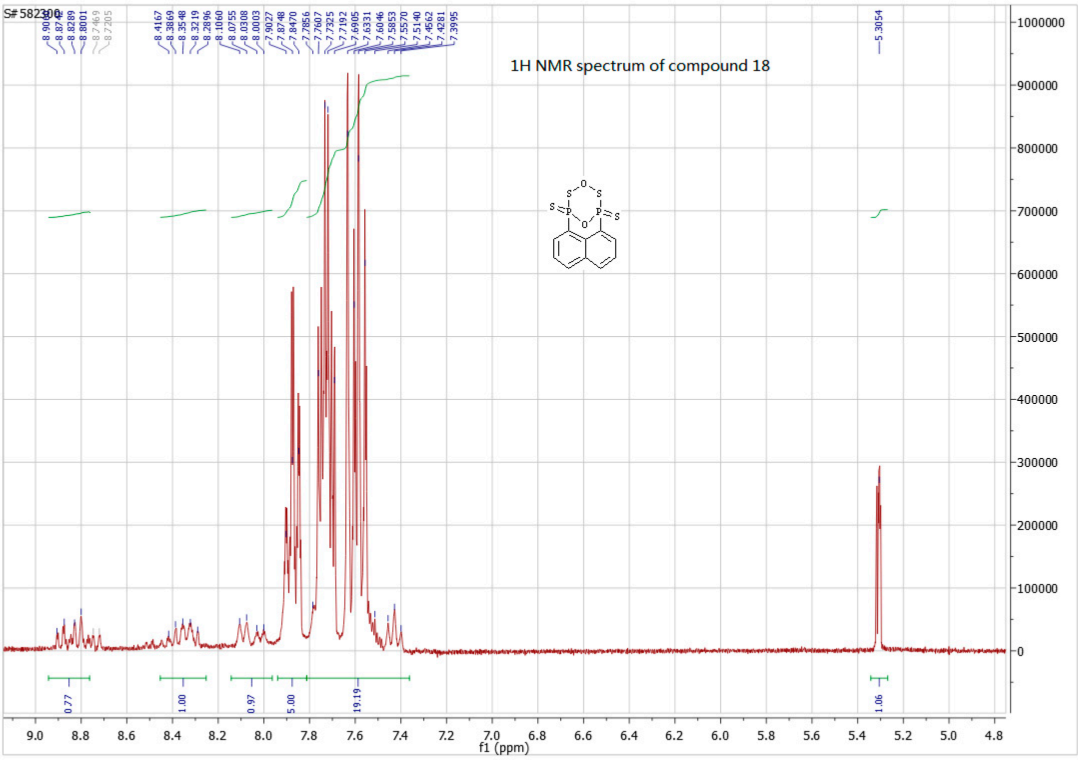


**Figure S29.** $^{1}$H-NMR spectra of compound **18**.

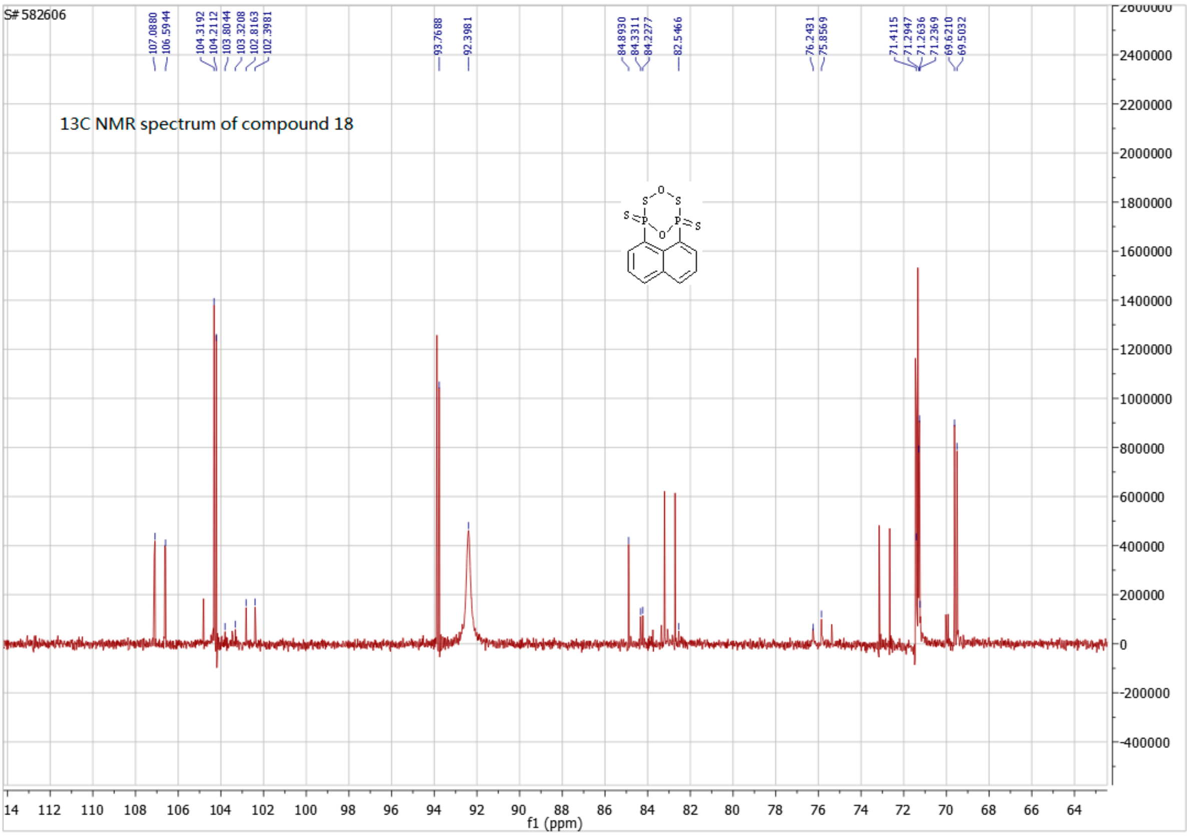


**Figure S30.** $^{13}$C-NMR spectra of compound **18**.

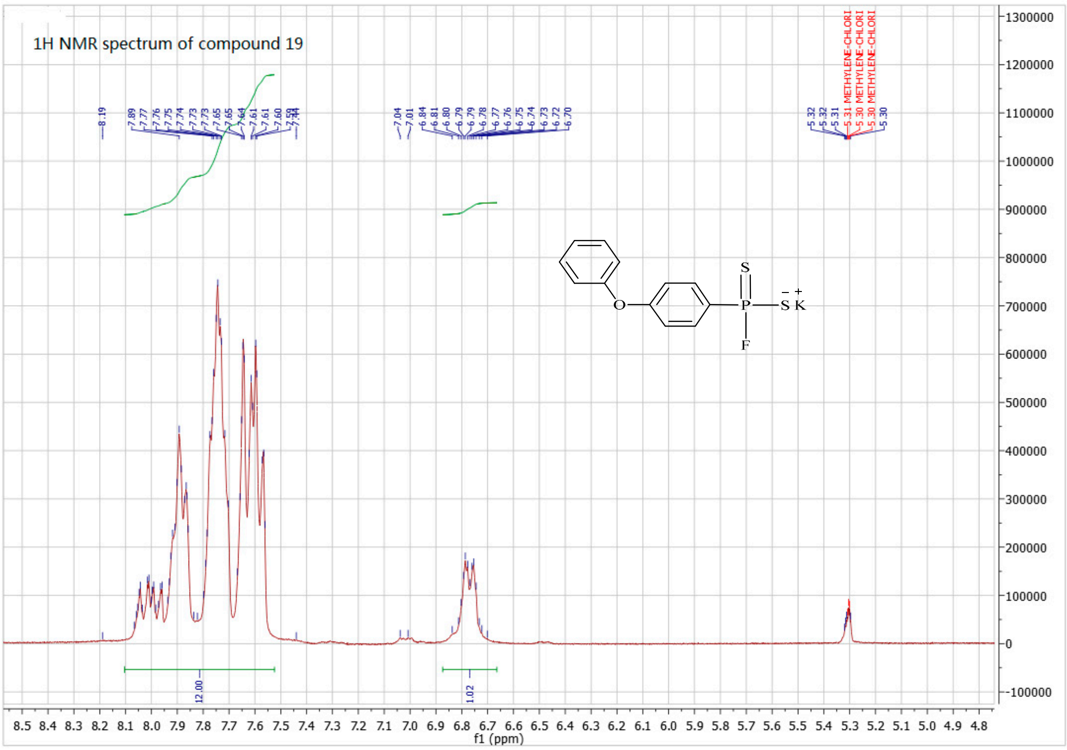


**Figure S31.** $^{1}$H-NMR spectra of compound **19**.

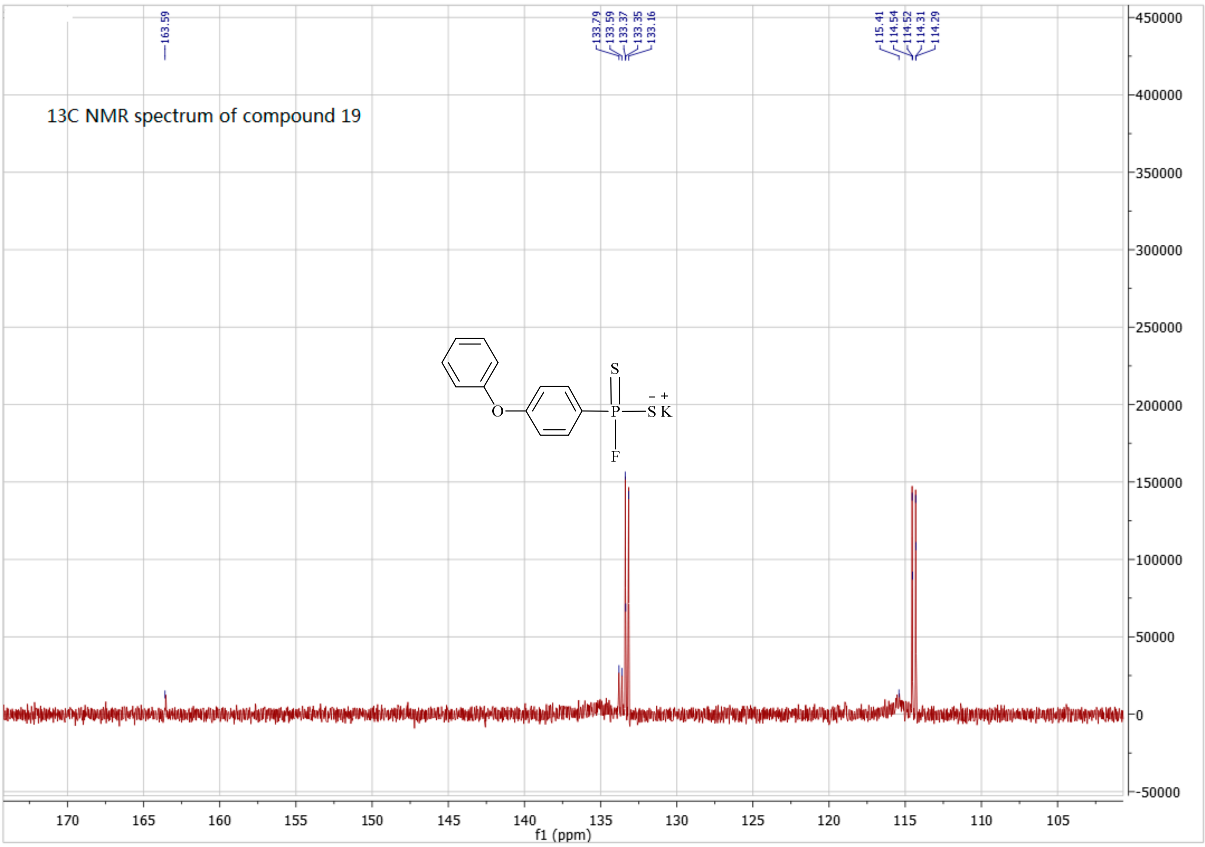


**Figure S32.** $^{13}$C-NMR spectra of compound **19**.

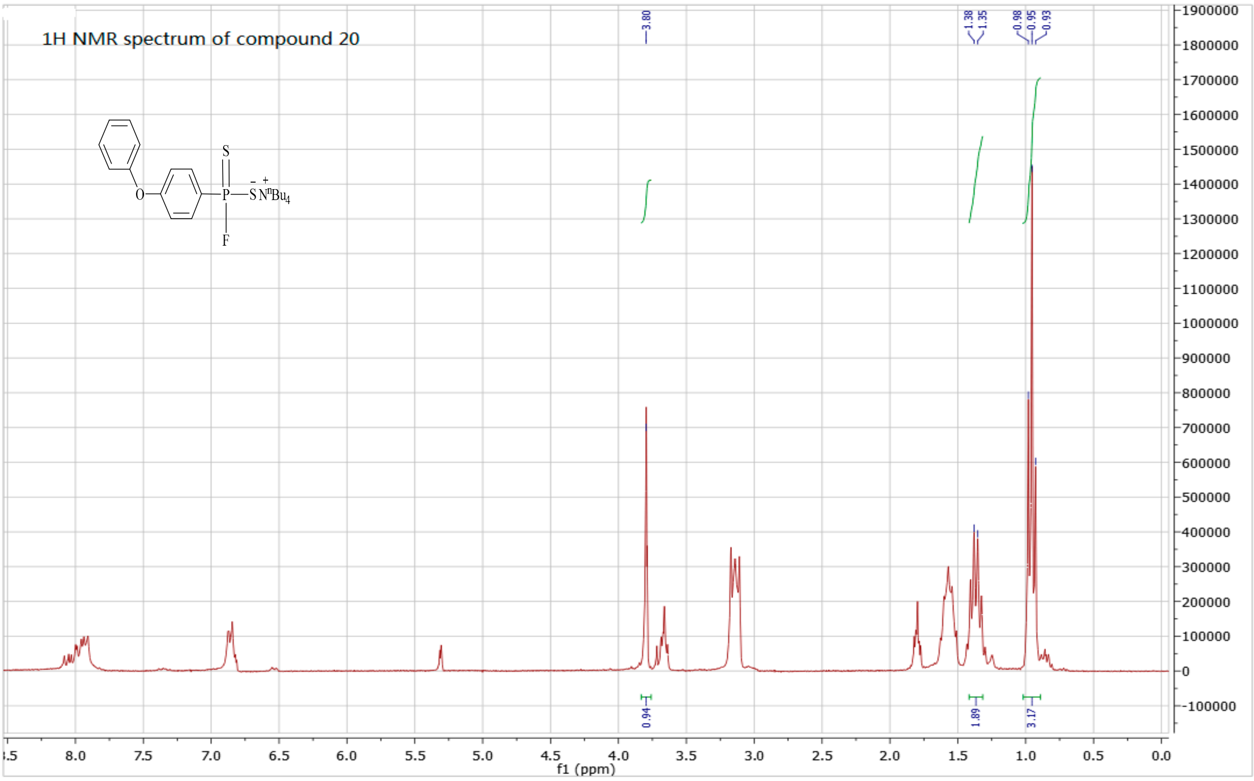


**Figure S33.** $^{1}$H-NMR spectra of compound **20**.

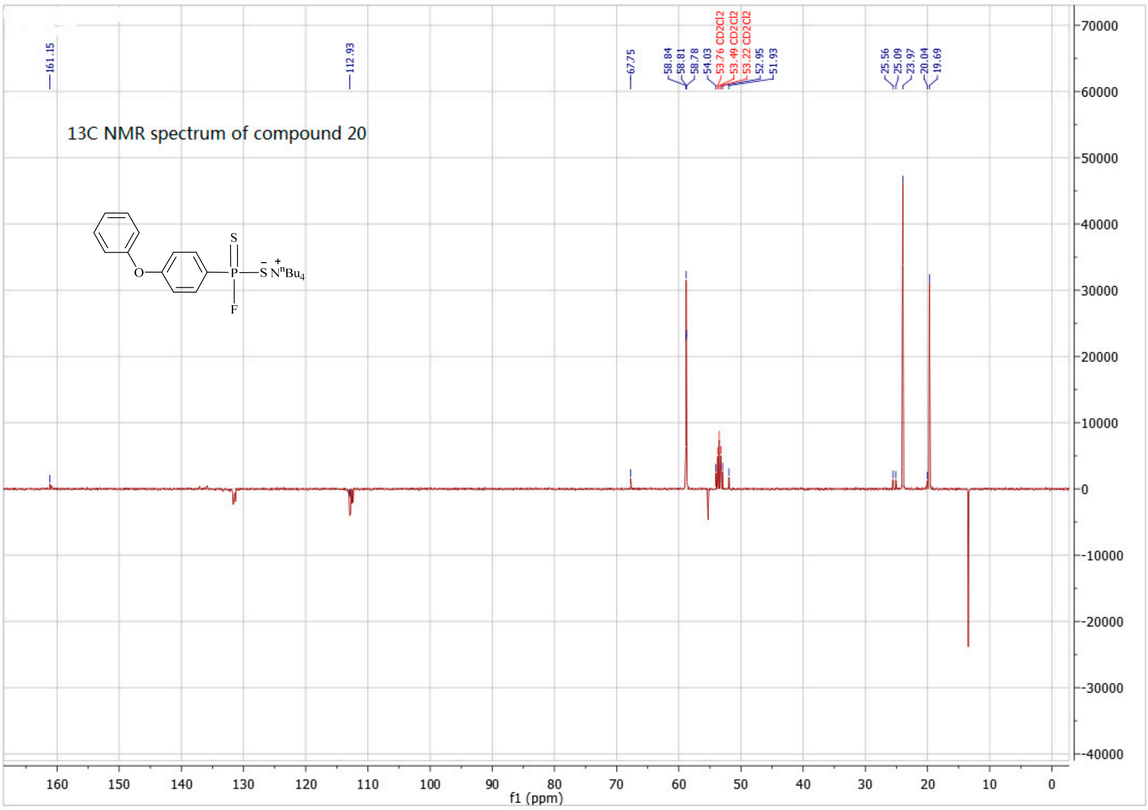


**Figure S34.** $^{13}$C-NMR spectra of compound **20**.

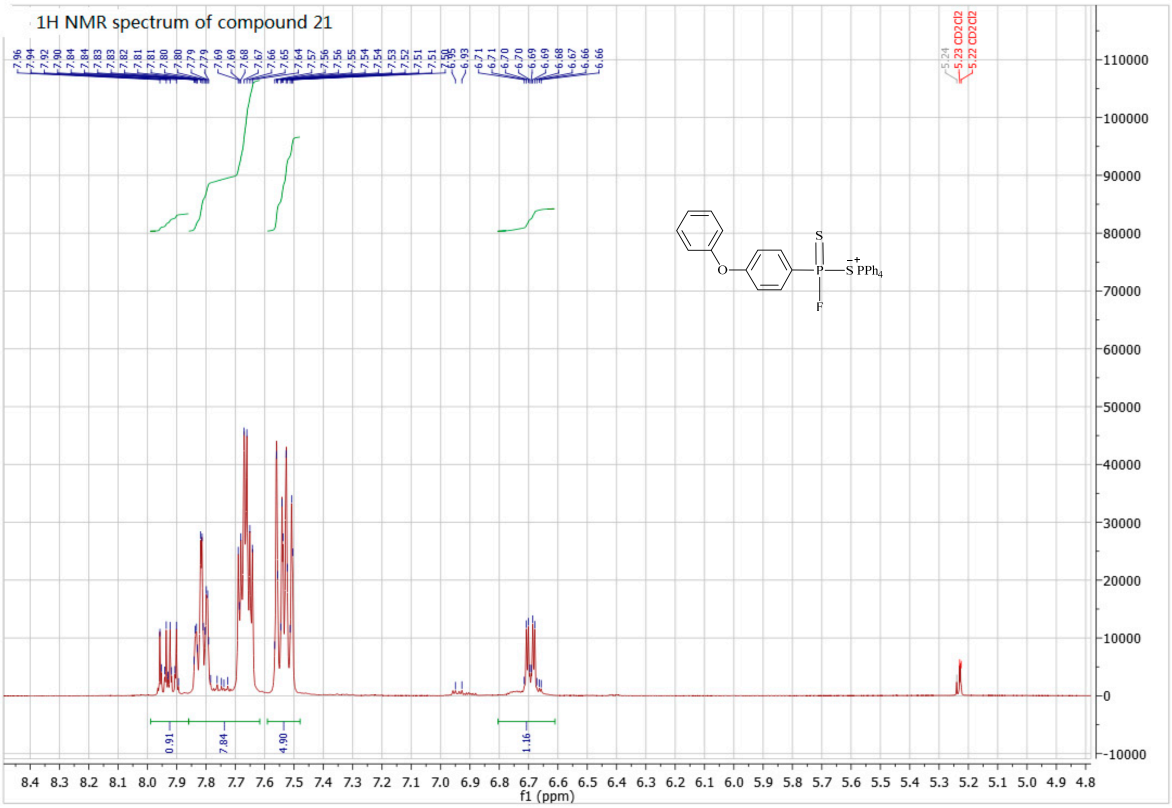


**Figure S35.** $^{1}$H-NMR spectra of compound **21**.

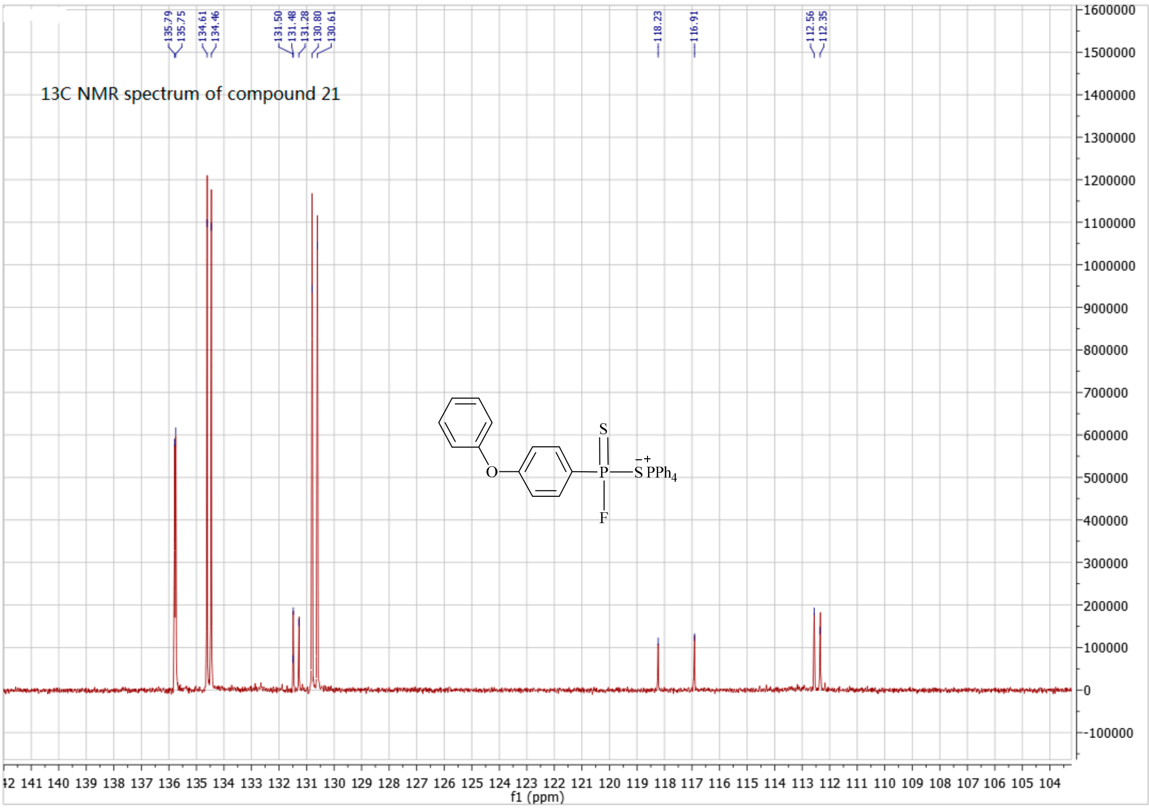


**Figure S36.** $^{13}$C-NMR spectra of compound **21**.
